# The Oviductal Extracellular Vesicles’ RNA Cargo Regulates the Bovine Embryonic Transcriptome

**DOI:** 10.3390/ijms21041303

**Published:** 2020-02-14

**Authors:** Stefan Bauersachs, Pascal Mermillod, Carmen Almiñana

**Affiliations:** 1 Genetics and Functional Genomics, VetSuisse Faculty Zurich, University of Zurich, 8315 Lindau (ZH), Switzerland; stefan.bauersachs@uzh.ch; 2 UMR85 PRC, INRA, CNRS 7247, Université de Tours, IFCE, 37380 Nouzilly, France; pascal.mermillod@inra.fr

**Keywords:** extracellular vesicles, exosomes, oviduct, embryo, gene expression, EV RNA cargo, EV-derived mRNAs, EV-derived miRNAs

## Abstract

Oviductal extracellular vesicles (oEVs) are emerging as key players in the gamete/embryo–oviduct interactions that contribute to successful pregnancy. Various positive effects of oEVs on gametes and early embryos have been found in vitro. To determine whether these effects are associated with changes of embryonic gene expression, the transcriptomes of embryos supplemented with bovine fresh (FeEVs) or frozen (FoEVs) oEVs during in vitro culture compared to controls without oEVs were analyzed by low-input RNA sequencing. Analysis of RNA-seq data revealed 221 differentially expressed genes (DEGs) between FoEV treatment and control, 67 DEGs for FeEV and FoEV treatments, and minor differences between FeEV treatment and control (28 DEGs). An integrative analysis of mRNAs and miRNAs contained in oEVs obtained in a previous study with embryonic mRNA alterations pointed to direct effects of oEV cargo on embryos (1) by increasing the concentration of delivered transcripts; (2) by translating delivered mRNAs to proteins that regulate embryonic gene expression; and (3) by oEV-derived miRNAs which downregulate embryonic mRNAs or modify gene expression in other ways. Our study provided the first high-throughput analysis of the embryonic transcriptome regulated by oEVs, increasing our knowledge on the impact of oEVs on the embryo and revealing the oEV RNA components that potentially regulate embryonic development.

## 1. Introduction

Extracellular vesicles (EVs) are well recognized mediators of cell-to-cell communication [1], a function they carry out by transferring their bioactive molecular cargo (RNAs, proteins, lipids, metabolites, and genomic DNA) to recipient cells [2,3]. Although at least three different types of EVs have been described based on their biogenesis and physical characteristics—exosomes, microvesicles and apoptotic vesicles [4]—only the first two types have attracted much attention in recent years, due to their contribution to a wide range of physiological and pathological processes such as angiogenesis, cell survival, modulation of the immune response, inflammation, and cancer, as well as embryonic development [5,6]. In fact, EVs identified in the oviduct and in the uterus have emerged as key players in the embryo–maternal dialogue contributing to successful pregnancy [7,8,9].

In particular, the potential role of oviductal EVs (oEVs) has received growing attention in recent years, since the oviduct is the place that hosts and supports the first reproductive events [10,11], and oEVs could be key modulators of such events. To date, EVs have been identified in the oviduct of different species (bovine, mouse, porcine, avian, and turtle) and their functional effects have been studied in gametes and embryos (reviewed in Almiñana and Bauersachs [12]). For example, it has been shown that oviductal EVs (oEVs) support bovine embryonic development [13,14], canine oocyte maturation [15], modulate sperm capacitation and sperm fertilizing ability in the mouse and in the cat [11,16], and regulate polyspermy fertilization in the pig [17]. Regarding the effects of oEVs on embryonic development, our laboratory previously demonstrated that oEVs are taken up by the bovine embryo during in vitro culture, and that the supplementation of oEVs during in vitro embryo culture improved embryonic development and quality in terms of blastocyst rates, cell number, and hatching rates [13]. Moreover, we showed that frozen and fresh oEVs had different effects on embryonic development and quality [13]. Along the same lines, Lopera-Vásquez [14] reported that oEVs enhanced embryo cryosurvival. Furthermore, Lopera-Vásquez [14,18] showed that oEV supplementation during in vitro culture altered the expression of a few genes involved in embryonic development, metabolism, and epigenetic regulation, making the embryos more similar to their in vivo counterparts [14]. These two studies by Lopera-Vásquez et al. [14,18] provided a few hints about the potential role of oEVs in modulating embryonic gene expression by using a targeted RT-qPCR approach, and called for an in-depth analysis of the impact of oEVs on the embryonic transcriptome.

Given the wide range of oEV components recently identified in our laboratory (mRNAs, proteins, ncRNAs including miRNAs, snoRNAs, snRNAs, and metabolites) [19,20], it is difficult to select potential candidates as modulators of embryonic development. To date, only a few miRNAs and proteins have been proven to be responsible for oEVs’ functional effects on spermatozoa [11,16], while the functional impact of the oEV cargo on embryos and the extent of those effects is not yet fully understood.

Therefore, in the present study we aimed to demonstrate that the RNA cargo in oEVs regulates early embryonic development by altering the embryonic transcriptome. We hypothesized that oEVs bring RNA components (mRNAs and miRNAs) and proteins into the embryo and thus alter the embryonic transcriptome. Moreover, we propose different modes of action by which the RNA cargo of oEVs could modify the embryonic transcriptome: (1) oEV-derived mRNAs could be incorporated into embryos via EVs and thereby increase the concentration of the delivered transcripts; (2) delivered mRNAs could be translated and the corresponding proteins could lead to regulation of embryonic gene expression; and (3) oEV-derived miRNAs and other ncRNAs could act by targeting embryonic mRNAs, and thus downregulate or modify embryonic gene expression in other ways (e.g., mRNA isoform expression, indirect effects on gene expression). In addition, given the differential effect of fresh and frozen oEVs on embryonic development, as demonstrated in our previous study [13], we also hypothesized that frozen and fresh oEVs affect the embryonic transcriptome differently. To this end, we used a low-input RNA-seq approach to profile the transcriptional responses of embryos cultured in vitro with fresh and frozen oEVs and controls without the addition of oEVs. Subsequently, to unveil the potential oEV RNA components capable of regulating the embryonic development, we performed an integrative analysis of mRNA and miRNA cargo identified in oEVs [19] and the embryonic transcriptome alterations induced by oEVs. The knowledge derived from our study will lead to a more meaningful understanding of the impact of oEVs on the embryo, while revealing the oEV RNA cargo potentially involved in the regulation of embryonic development.

## 2. Results

### 2.1. Oviductal EV Supplementation during In Vitro Embryo Culture Altered the Embryonic Transcriptome

The oEVs used in this study were derived from cows in the postovulatory stage. In our previous studies, characterization of these oEVs revealed a population of small extracellular vesicles (30–100 nm) resembling exosomes (50%–60% of all vesicles) and a population of larger extracellular vesicles (>100 nm) resembling microvesicles (25%–30% 100–150 nm, 10% 150–200 nm) [13,19]. Typical EV marker proteins were detected by Western blotting, such as HSP70, ANXA1, MYH9, and HSPA8 [13,19]. In the present study, we focused on analysis of the transcriptome of embryos cultured in vitro with or without oEV supplementation.

**Figure 1:**
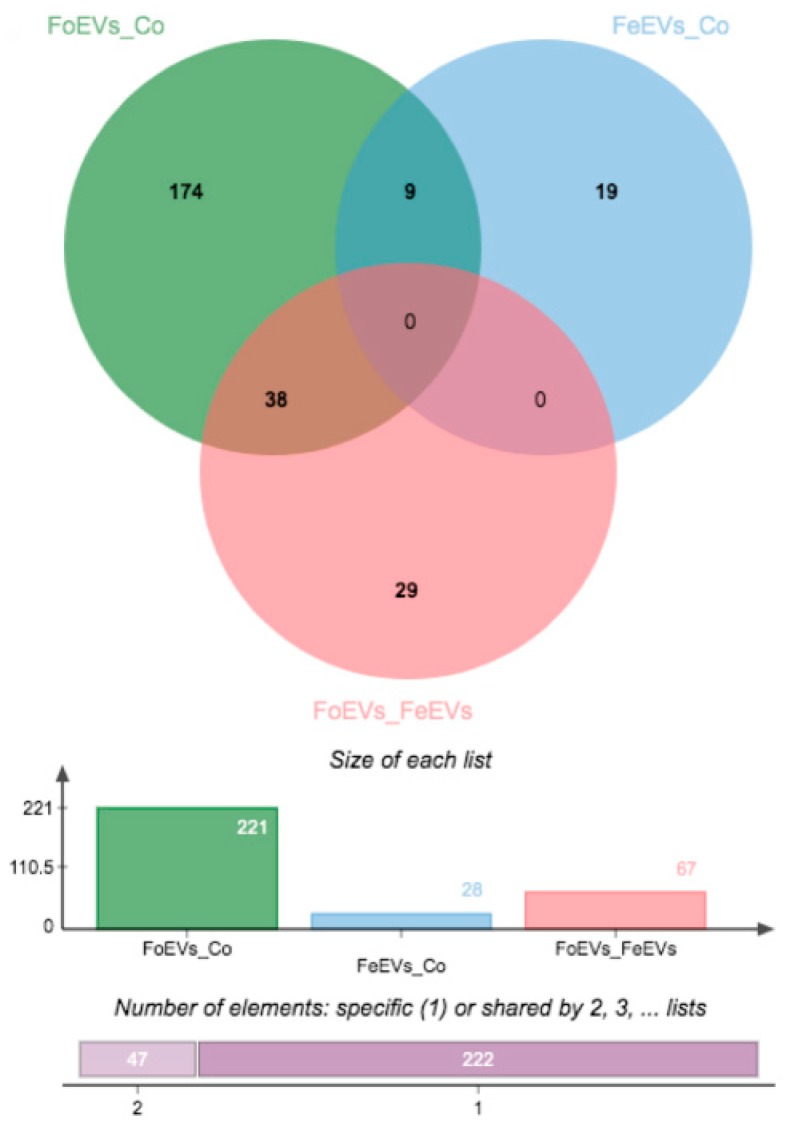
Venn diagrams showing the distribution of differential expressed genes (DEGs) regulated by oviductal extracellular vesicle (oEV) treatments. Data were based on a *p*-value cut-off of 0.001 and an FDR of 0.05 (FoEV treatment vs. Co). FoEVs_Co: embryos cultured with frozen oEVs (FoEVs) compared to embryos without oEVs (Co, control); FeEVs_Co: embryos cultured with fresh oEVs (FeEVs) compared to Co; FoEVs_FeEVs: FoEVs compared to FeEVs.

We hypothesized that oEVs bring RNA components (mRNAs and miRNAs) into the embryo, which then alter its transcriptome. Moreover, we hypothesized that frozen and fresh oEVs affect the embryonic transcriptome differently. To prove our hypothesis, RNA-seq analysis of embryos cultured in vitro with frozen oEVs (FoEVs) and fresh oEVs (FeEVs) and without oEVs (Co, control) was performed. Transcripts derived from a total number of 10,832 genes were identified in all embryos examined under different in vitro culture (IVC) treatments (after filtering for a minimum number of read counts; Supplementary Data S1(Table S1)). To identify genes with altered gene expression due to different IVC treatments, statistical analysis was performed between FoEV treatment and Co, FeEV treatment and Co, and between both EV treatments (FoEVs and FoEVs). The total number of differentially expressed genes (DEGs) for these comparisons was 316, based on a false discovery rate (FDR) < 0.05 for the comparison FoEV treatment vs. Co (Supplementary Data S1(Tables S2–S4)). The overlap of DEGs among comparisons is shown in a Venn diagram (Figure 1).

**Figure 2:**
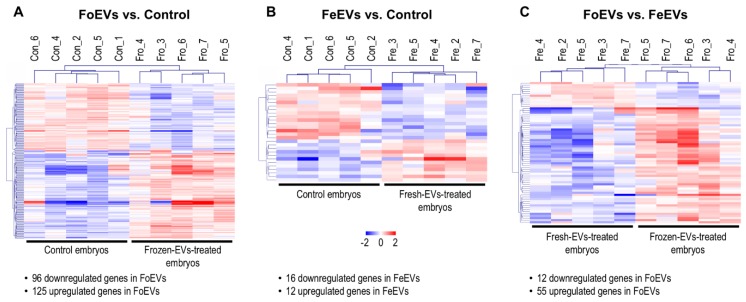
Comparative transcriptome analysis of embryos cultured in vitro under different treatments; (**A**) FoEV-treated and Co embryos; (**B**) FeEV-treated and Co embryos; (**C**) FoEV- and FeEV-treated embryos (Co: control; FoEV: frozen EV; FeEV: fresh EV). Dendrograms representing results of unsupervised hierarchical clustering (HCL) created with Pearson correlation coefficient by MeV. Rows show DEGs while columns represent embryo samples collected under different IVC treatments. Each sample represents a pool of embryos. Mean-centered expression values (log2 counts per million of sample-mean of log2 CPM of all samples) for the samples of the three embryo treatments. Color scale is from -2 (blue, lower than mean) to 2 (red, higher than mean).

Hierarchical cluster analysis of the DEGs obtained for the three comparisons is shown in Figure 2. The main differences were identified between FoEV treatment and Co (221 DEGs; Figure 1 and Figure 2A), the lowest for the comparison between FeEV and Co treatments (28 DEGs; Figure 1 and Figure 2B), and 67 DEGs for the comparison between EV treatments (Figure 1 and Figure 2C). Furthermore, when an additional cut-off was considered (minimum fold change of 1.5), 112, 19, and 46 DEGs were obtained for comparisons between FoEV treatment and Co, FeEV treatment and Co, and between EV treatments, respectively (Supplementary Data S1(Tables S2–S4)).

Among the 221 DEGs between FoEV-treated and control embryos, 96 were downregulated and 125 upregulated in FoEV-supplemented embryos compared to control embryos. Of the 125 mRNAs upregulated in FoEV-treated embryos compared to control, 20% (25) were annotated as different types of small non-coding RNAs (sncRNAs). Among them, 12 were identified as bovine small nucleolar RNA, C/D (known as SNORDs, human symbols: SNORD10, SNORD11, SNORD13, SNORD15A, SNORD15B, SNORD21, SNORD27, SNORD46, SNORD53B, SNORD70, SNORD103B); 7 as bovine small nucleolar RNA, H/ACA (known as SNORA, human symbols: SNORA16A, SNORA23, SNORA46, SNORA54, SNORA63, SNORA73A, SNHG25), and 6 as small nuclear RNAs which play important roles in RNA splicing (human symbols: RNU1-1, RNU1-13P, RNU2-1, RNU4-2, RNU1-1-like, RNU4-2-like). Among the 28 DEGs in FeEV-treated embryos, 16 were downregulated and 12 upregulated upon FeEV treatment compared to control. Finally, among 67 DEGs between FoEV- and FeEV-treated embryos, 12 were downregulated and 55 upregulated upon FoEV treatment. The complete lists of down- and upregulated genes for each comparison can be found in Supplementary Data S1.

### 2.2. Functional Annotation Revealed Potential Regulatory Pathways Affected by Oviductal EVs

To obtain a more meaningful view of how oEVs can modulate the embryo transcriptome, gene ontology terms (GO) terms and pathways for DEGs from the three different comparisons (FoEV treatment vs. Co; FeEV treatment vs. Co, and FoEV vs. FeEv treatment) were analyzed using functional annotation databases in Metascape and with Ingenuity Pathway Analysis (IPA) tools.

**Figure 3:**
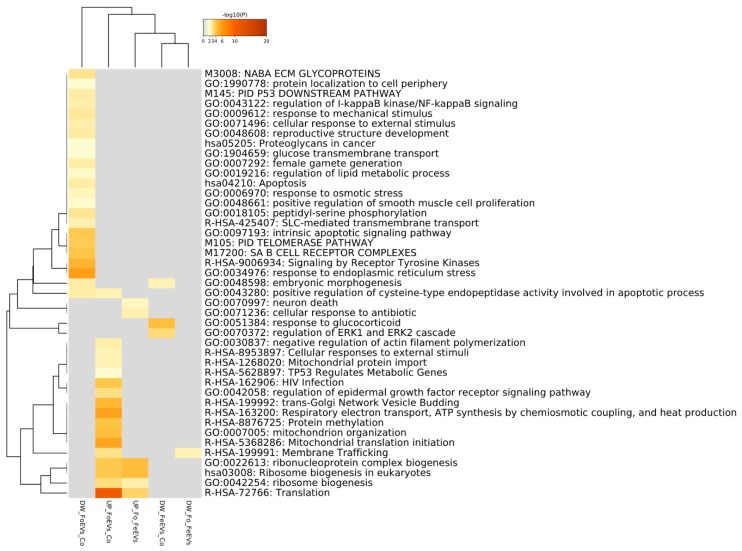
Functional enrichment analysis for up- and downregulated genes in embryos treated with FoEVs or FeEVs compared to control and between oEV treatments using Metascape tool. Bar graph of enriched terms across DEGs colored by *p*-values representing enriched clusters up to a score of 2.

In Figure 3, a Metascape heatmap plot represents the functional enrichment analysis for up- and downregulated genes in embryos treated with frozen or fresh EVs compared to control and between EV treatments, and shows clusters of enriched functional terms across DEGs from different comparisons. The heatmap illustrates that functional terms and pathways related to “embryo morphogenesis” were only enriched for genes downregulated in FoEV treatment vs. Co and FeEV treatment vs. Co sets. Furthermore, genes downregulated in FoEV treatment vs. Co were also enriched for biological processes related to “female gamete generation”, “apoptosis”, “response to external stimulus”, “response to osmotic stress”, “telomerase pathway”, “glucose transmembrane transport”, and “regulation of lipid metabolic process”. For the upregulated genes in the FoEV treatment vs. Co comparison, we found high enrichment of “translation” and also of functional terms involved in “ribosome biogenesis”, “membrane trafficking”, “mitochondrial organization”, and “protein methylation”. Additionally, FoEV treatment vs. Co upregulated genes were involved in “negative regulation of actin filament polymerization”. When both EV treatments were compared, the GO terms found to be enriched were “translation”, “ribonucleoprotein complex biogenesis”, and “ribosome biogenesis” for upregulated genes, and “membrane trafficking” for downregulated genes in the FoEV treatment vs. FeEV treatment comparison.

More details for the obtained overrepresented functional terms and pathways for each DEG set obtained from embryos under different EV treatments compared to control, with highlighted genes related to interesting biological processes and pathways can be found summarized in Table 1, Table 2, Table 3, Table 4, Table 5 and Table 6 and in Supplementary Data S2(Tables S1–S6).

**Figure 4:**
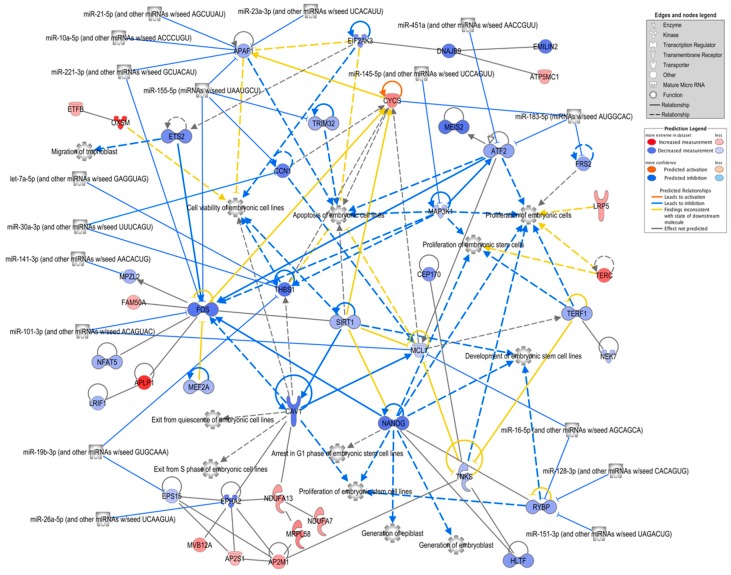
Network of FoEVs vs. Co DEGs involved in embryonic development. A network of overrepresented biological functions related to embryonic development and their assigned FoEV-treated embryos vs. control DEGs was generated using ingenuity pathway analysis software. MicroRNAs revealed by the software to be potential upstream regulators were added to the network (restricted to miRNAs known to be present in bovine oEVs) as well as further connected DEGs. Genes are colored by log2 fold change ratio, blue for downregulated and red for upregulated genes.

The analysis of the DEG lists performed with IPA software (gene expression core analysis) revealed basically similar results, i.e., similar overrepresented functional terms and pathways as were obtained with Metascape. Thus, the analysis was focused on genes related to embryonic development by searching for all enriched related functions and generating a network of the associated genes, including 17 miRNAs potentially targeting the genes in the network and previously identified in bovine oEVs [19] (Figure 4; Supplementary Data S2(Tables S9 and S10)). Overall, the majority of the assigned genes had lower mRNA levels in the embryos supplemented with frozen oEVs. The genes with the highest connectivity were Nanog homeobox (*NANOG*), MCL1 apoptosis regulator, BCL2 family member (*MCL1*), Fos proto-oncogene, AP-1 transcription factor subunit (*FOS*), activating transcription factor 2 (*ATF2*), apoptotic peptidase activating factor 1 (*APAF1*), cytochrome c, somatic (*CYCS*), and thrombospondin 1 (*THBS1*). The processes with the highest connectivity were “apoptosis of embryonic cell lines”, “proliferation of embryonic cells”, and “cell viability of embryonic cell lines”. The genes targeted by the highest number of miRNAs were *APAF1* (five miRNAs), *FOS* (three miRNAs), *THBS1* (three miRNAs), and RING1 and YY1 binding protein (*RYBP*, three miRNAs).

### 2.3. Comparative and Integrative Analysis of Embryonic mRNAs Altered under oEV Treatment and oEV-Derived mRNAs

**Figure 5:**
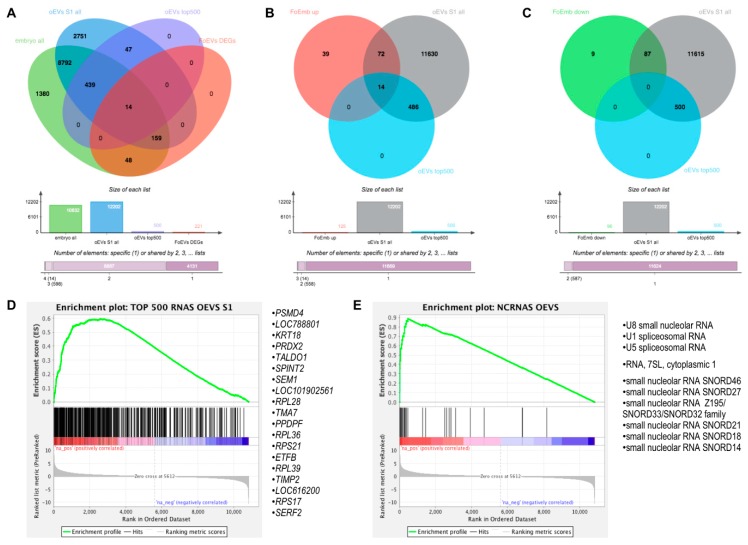
Comparative analysis of embryonic mRNAs altered under oEV treatment and oEV-derived mRNAs and ncRNAs. (**A**) Venn diagrams representing the comparison among all identified mRNAs in embryos, upregulated DEGs in embryos supplemented with frozen EVs (FoEVs) compared to control embryos, all mRNAs in oviductal EVs (oEVs), and top 500 most abundant mRNAs in oEVs; (**B**) comparison among upregulated DEGs in FoEV treatment vs. Co, all mRNAs in oEVs, and the top 500 most abundant mRNAs in oEVs; (**C**) comparison among downregulated DEGs in DEGs in FoEV-treated vs. Co embryos, all mRNAs in oEVs, and the top 500 most abundant mRNAs in oEVs; (**D**) gene set enrichment analysis (GSEA) showing enrichment of the top 500 most abundant mRNAs in oEVs in the rank list of all genes detectable in embryos supplemented with frozen oviductal EVs (oEVs) ranked by differential expression (red = higher expression in FoEVs-treated embryos compared to control embryos, blue = lower expression), and the 19 genes with the highest enrichment scores; (**E**) GSEA enrichment plot showing enrichment of oEVs-derived non-coding RNAs (ncRNAs) in the rank list (same list as in D), and the 10 ncRNAs with the highest enrichment scores.

To unveil the oEV mRNA cargo responsible for the embryonic transcriptomic changes observed in the present study, we compared the mRNAs identified in oEVs in our previous study [19] at the post-ovulatory stage (S1) and the embryonic mRNAs identified under oEV treatments (from the post-ovulatory stage in FoEV-treated embryos) from the present study. The Venn diagram in Figure 5A shows a comparison of all detectable genes in the embryos (Supplementary Data S1(Table S1)), the DEGs for the comparison FoEV treatment vs. Co (Supplementary Data S1(Table S2)), all detectable genes in the postovulatory-stage oEVs (Supplementary Data S1(Table S7)), and the top 500 genes with highest expression in postovulatory-stage oEVs (based on transcripts per million, TPM; Supplementary Data S1(Table S8)). The comparison of all detectable genes revealed an overlap of 9404 genes. Considering that the transcripts with low concentrations in oEVs might not be very likely to have an effect on the embryo, we selected the top 500 most abundant transcripts that showed a frequency of more than 25 TPM [19], resulting in 453 embryonic mRNAs in total and 14 of the DEGs of the FoEV treatment vs. Co comparison (Supplementary Data S1(Table S9)) as being in common with the top 500 mRNAs in oEVs.

To further test the first of our hypotheses, that a part of the alterations of the embryonic transcriptome could be directly due to RNAs derived from oEVs that simply increase the number of these transcripts in the embryo due to oEV delivery, we compared upregulated DEGs in frozen-EV-derived embryos compared to control (125 genes, Supplementary Data S1(Table S5)) to all mRNAs in oEVs and the top 500 most abundant mRNAs in oEVs (Figure 5B). This comparison revealed 86 upregulated genes in common with all oEV mRNAs (Supplementary Data S1(Table S9)) and 14 upregulated genes in common with the top 500 most abundant mRNAs in oEVs (Supplementary Data S1(Table S9)). In contrast, the genes downregulated in frozen-EV-supplemented embryos (96 genes, Supplementary Data S1(Table S6)) did not overlap with the top 500 most abundant mRNAs in oEVs (Figure 5C), whereas a list of 87 genes in common with all mRNAs in oEVs was obtained (Supplementary Data S1(Table S9)).

Furthermore, we used gene set enrichment analysis (GSEA) [21] to identify significantly enriched genes in embryos which might be derived from oEVs by comparison to (1) the top 500 genes with the highest expression in oEVs and (2) non-coding RNAs contained in oEVs (Supplementary Data S1(Table S10)) [19]. The GSEA plot in Figure 5D shows a substantial enrichment of the top 500 most abundant mRNAs identified in oEVs towards the upregulated genes in oEV-supplemented embryos. The list of the 19 genes with the highest enrichment scores obtained from this analysis is also shown in Figure 5D. Moreover, a strong enrichment was also found for a group of ncRNAs identified in oEVs containing mainly small nucleolar RNAs (snoRNAs) and spliceosomal RNAs, as illustrated in Figure 5E. The complete lists of GSEA results can be found in the Supplementary Data S1(Tables S11 and S12). Comparison of Venn diagram and GSEA results revealed a set of 14 genes common to both analyses as potential mRNAs contained in oEVs contributing to upregulation of embryonic gene expression, as well as a set of small non-coding RNAs, including snoRNAs and spliceosomal RNAs.

Moreover, transcripts delivered by oEVs to the embryo could be translated and regulate gene expression in the embryo. To identify potential factors upregulating embryonic gene expression, the upregulated genes in FoEV-treated embryos were subjected to transcription factor (TF) analysis using ChIP-X Enrichment Analysis Version 3 (ChEA3) [22]. This analysis revealed five factors, HMGN3, JUND, NME2, PIN1, and YBX1, of which the mRNAs were contained in the top 500 most highly abundant mRNAs in oEVs, potentially regulating 53 of the upregulated genes (Supplementary Data S1(Table S13)).

### 2.4. Comparative Analysis of Embryonic mRNAs Altered under oEV Treatment and Potential Genes Targeted by oEV-Derived miRNAs

To test the second of our hypotheses, that the alterations of the embryonic transcriptome could also be due to miRNAs contained in oEVs that downregulate mRNAs in the embryo, we used three different approaches.

**Figure 6:**
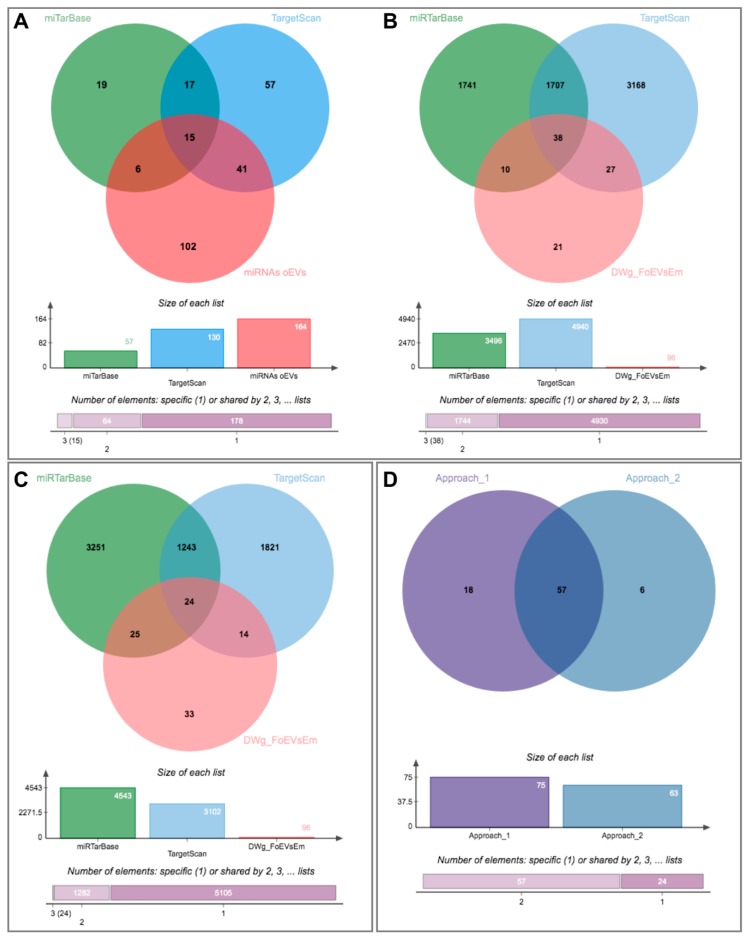
Identification of potential miRNAs in oviductal EVs responsible for downregulation of embryo gene expression using two different approaches: (1) From identified downregulated genes in embryos to potential miRNAs in oEVs and (2) From identified miRNAs in oEVs to potential target genes in embryos. (**A**) Venn diagram representing the comparison among potential miRNAs targeting identified downregulated genes in embryos using miTarBase and TargetScan databases and identified miRNAs in oEVs; (**B**) Venn diagram representing the comparison of potential target genes of the miRNAs derived from the previous comparison and identified downregulated genes in embryos; (**C**) Venn diagram representing the comparison among potential target genes derived from identified miRNAs in oEVs using miTarBase and TargetScan databases and identified downregulated genes in embryos; (**D**) comparison of lists of target genes from both approaches.

The first approach was based on identifying potential miRNAs that could target the identified downregulated genes in embryos treated with oEVs and comparing them to identified miRNAs in oEVs. Figure 6A represents the comparison of potential miRNAs targeting identified downregulated genes in embryos using miTarBase and TargetScan databases to the miRNAs identified in oEVs (62 miRNAs, Supplementary Data S1(Table S14)). Based on the overlapping miRNAs, potential target genes derived from miTarBase and TargetScan datasets were compared to identified downregulated genes in embryos (Figure 6B, Supplementary Data S1(Table S14)). This first approach provided a list of 75 predicted target mRNAs of miRNAs contained in oEVs common to genes downregulated in embryos supplemented with FoEVs.

The second approach was based on identifying potential target genes of the miRNAs identified in oEVs and comparing them to the observed downregulated genes in embryos treated with oEVs. The Venn diagram illustrated in Figure 6C shows the results of this comparison, representing potential target genes derived from miTarBase and TargetScan databases and the downregulated genes identified in embryos (Supplementary Data S1(Table S14)). This second approach provided a list of 63 predicted targets of miRNAs in oEVs and common to downregulated genes in embryos supplemented with FoEVs. A comparison of the lists of target genes derived from the two approaches provided a list of 57 commonly identified genes, of which expression is probably downregulated in frozen-oEV-supplemented embryos due to miRNAs derived from oEVs (Figure 6D, Supplementary Data S1(Table S14)).

Since we found genes downregulated in frozen-oEV-supplemented embryos to be mainly associated with functions such as embryonic development, embryo death, embryonic pluripotent cell lines etc. in the IPA analysis (Figure 4), we looked for potential activated upstream regulators and selected significant miRNAs that were then added to the network. Seventeen miRNAs identified in oEVs were connected to potential targets in the network according to the IPA knowledge base. Fifteen of these miRNAs were also contained in the 62 miRNAs potentially targeting identified downregulated genes in embryos using the miTarBase and TargetScan databases (Figure 6A).

### 2.5. Integrative Analysis of mRNAs and miRNAs Contained in oEVs and Embryonic Transcriptome Alterations Induced by oEVs

**Figure 7:**
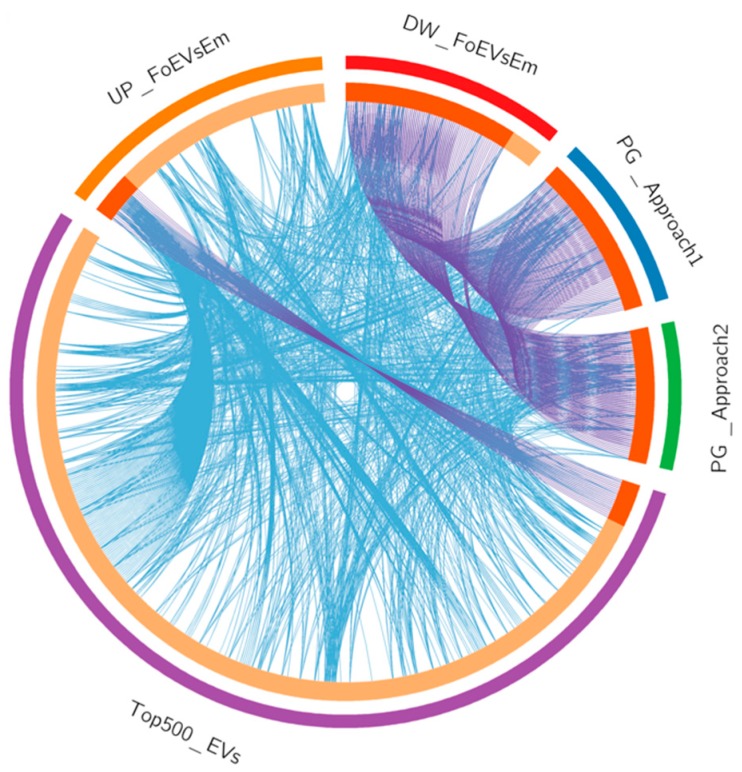
Meta-enrichment analysis summary for list of identified and potential target genes in embryos regulated by miRNAs in oEVs. Input lists: (1) the top 500 mRNAs in oEVs (Top500_EVs, Purple); (2) the upregulated genes in FoEV-treated versus Co embryos (UP_FoEVsEmb, Orange); (3) the downregulated genes in FoEV-treated versus Co embryos (DW_FoEVsEmb, Red); (4) the potential genes targeted by miRNAs contained in oEVs based on Approach 1 from identified downregulated genes (PG_miRNAs_Evs_T1, Blue); and (5) based on Approach 2 from identified miRNAs in oEVs (PG_miRNAs_Evs_T2, Green). Circular plot representing overlaps among gene lists at the gene level, where purple lines link identical genes and blue lines link genes belonging to the same enriched ontology term. The inner circle represents gene lists, where hits are arranged along the arc. Genes hit multiple lists are colored in dark orange, and genes unique to a list are shown in light orange.

Datasets of oEV mRNAs (top 500) and predicted targets of oEV miRNA cargo as well as DEGs in embryos in response to FoEVs were plotted in a circular layout (Figure 7 and Supplementary Data S1(Table S15)), providing an integrative view of embryonic gene expression data and RNAs derived from oEVs and their potential effects on embryonic gene expression. Figure 7 shows the overlaps among gene lists at the gene level. This meta-enrichment summary analysis illustrated the two hypotheses postulated in the present study: (1) mRNAs derived from oEVs increase concentrations of a proportion of mRNAs in embryos treated with oEVs, which was supported by the fact that a considerable proportion of DEGs that were upregulated in embryos supplemented with frozen oEVs were present in the top 500 mRNAs in oEVs, and no overlap was found with genes downregulated in supplemented embryos; and (2) miRNAs derived from oEVs downregulate mRNAs in embryos treated with oEVs, which was supported by the overlap of the genes downregulated in embryos supplemented with frozen oEVs (DW_FoEVsEmb) and the genes predicted as targets of oEV miRNAs (PG_miRNA_EVs_T1 and PG_miRNA_EVs_T2).

**Figure 8:**
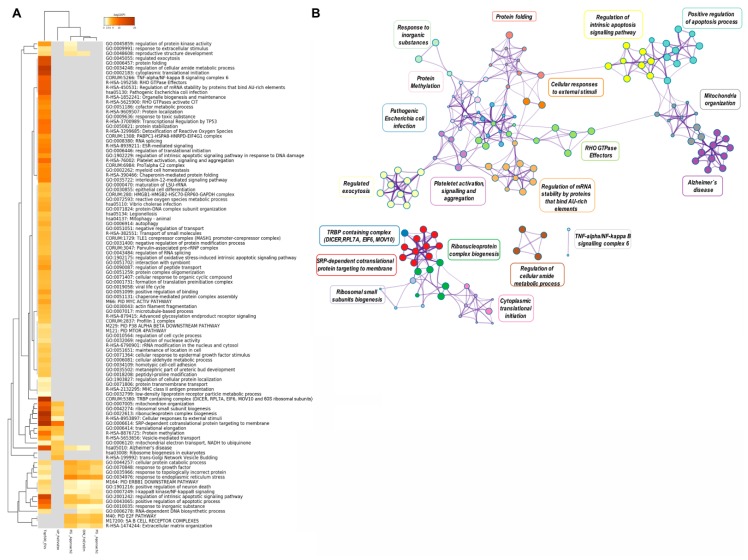
(**A**) Metascape heatmap of enriched terms across input gene lists representing the top 100 functional clusters: (1) the top 500 mRNAs in oEVs, (2) the upregulated genes in FoEV treatment vs. Co, (3) the downregulated genes in FoEV-treated vs. Co embryos, (4) the potential genes targeted by miRNAs in oEVs based on Approach 1 from the identified downregulated genes, and (5) based on Approach 2 from identified miRNAs in oEVs, all colored by *p*-values. (**B**) Metascape network of enriched terms and protein–protein interaction enrichment analysis of the same input lists. Pathway/cluster IDs shared across multiple lists have the same colors and nodes sharing the same cluster ID are closer to one another.

Further functional annotation analysis of this integrative data was represented in a heatmap of enriched terms across input gene lists (Figure 8A, Supplementary Data S2(Tables S7 and S8)). Interestingly, functional terms related to “mitochondria organization”, “ribosome biogenesis”, “ribonucleoprotein complex biogenesis”, “cellular response to external stimuli”, “SRP-dependent co-translational protein targeting to membrane”, “protein methylation”, and “vesicle-mediated transport” were highly enriched for the upregulated genes in embryos supplemented with frozen oEVs and the top 500 oEV mRNA datasets, while functional terms involved in “response to inorganic substances”, “cellular protein catabolic process”, “response to endoplasmic reticulum stress”, “regulation of intrinsic apoptosis signaling pathways”, and “positive regulation of apoptotic process” were highly enriched in downregulated genes (frozen oEV embryo treatment), predicted target genes derived for miRNAs in oEVs, and the top 500 oEV mRNA datasets. Interestingly, functional terms related to “reproductive structure development” were highly enriched in downregulated genes (frozen oEV embryo treatment), predicted target genes derived for miRNAs in oEVs, and the top 500 oEV mRNA datasets. Figure 8B provides a Metascape network of these enriched terms and the protein–protein interaction enrichment analysis.

## 3. Discussion

This study demonstrated that the supplementation of IVC media with oEVs alters the embryonic transcriptome. Moreover, differences in embryonic gene expression were found between embryos supplemented with oEVs that were frozen before adding to IVC (FoEVs) and with fresh oEVs (FeEVs). In the following sections, we discuss these different effects of FoEVs and FeEVs on the embryo transcriptome, which supported the differential functional changes observed in embryonic development when FeEVs and FoEVs were used in a previous study [13]. Moreover, we discuss different modes of action through which the oEV RNA cargo could regulate the embryo transcriptome and embryonic development by providing different modes of integrative analysis of data derived from our studies.

### 3.1. Differential Effect of Fresh and Frozen oEVs on Embryonic Transcriptome

The differential functional effects of fresh and frozen oEVs on in vitro embryonic development, as demonstrated in on our previous study [13], were reflected in the observed changes in the embryonic transcriptome in the present study. It is worth mentioning here that both oEV treatments were obtained from the same oviducts and split into two samples for FoEV and FeEV treatments. Thus, the only difference in the oEVs used for IVC supplementation was the freeze–thaw step for the FoEV samples. Studies on the effect of storage of EVs at −80 °C have shown controversial results. While some studies have indicated that storing EV samples at −80 °C does not alter EV morphology or size [23,24], others have demonstrated that EV integrity can be disrupted by freezing–thawing [25,26]. Boch and colleagues [25] evaluated the impact of one and four freeze–thaw steps on EV damage and showed that no measurable differences in the particle size distribution and concentration measured by nanoparticle tracking analysis (NTA) were observed after the first freeze–thaw step. However, four freeze–thaw cycles induced a slight increase in the particle size distribution and particle number for EVs stored in PBS in contrast to EVs stored in Trehalose, a protein stabilizer and cryoprotectant widely used in the food and pharmaceutical industries. We hypothesized that freezing might induce membrane alterations in FoEVs and faster leaking or release of EV content compared to FeEVs, thus resulting in a better transfer of the cargo to target cells. The study by Teng et al. (2015) [27] supported our hypothesis, showing a significant reduction in the bi-layer membrane of frozen vesicles (−80 °C) when compared to fresh EVs, even when only imperceptible changes of size and concentration were observed in EVs after one step of freezing–thawing [25]. Moreover, Maroto et al. (2016) [26] showed that in exosome preparations stored at −80 °C, proteins appeared in the supernatant fraction, suggesting that distinct protein groups leak from exosomes even at a −80 °C storage temperature. On the other hand, studies on EV cargo have shown that freezing seems to almost completely preserve the EV-associated proteins [28] and does not impair their functionality [23,29], as we observed in our study. It is worth noting that most EV functional studies have been based on frozen EVs for practical reasons, or due to the impossibility of performing functional studies immediately after EV isolation and characterization. However, not many studies have compared the functional effects of frozen and fresh EVs on cells to date. Further studies are required to elucidate the impact of the freezing process on EVs’ integrity, cargo, and functional effects, and the use of protective substances such as Trehalose. Nevertheless, the results from our laboratory provide clear evidence of the positive effect of fresh and frozen oEVs on embryonic development [13], and the extent of such effects to inducing modifications in embryonic development.

### 3.2. Oviductal EVs Regulate Early Embryonic Development by Altering the Embryonic Transcriptome

To date, our study represents the first high-throughput analysis of the effects of oEVs on the embryonic transcriptome. Two previous studies using a targeted approach by RT-qPCR showed that in vitro oEV supplementation induced few changes in gene expression related to epigenetic alterations, metabolism, and embryonic development [14,18]. Five genes, *PAG1*, *AQP3*, *LDLR*, *DNMT3A*, and *SNRPN*, were found to be upregulated in embryos when oEVs or isthmus-derived oEVs were used in in vitro embryo culture compared to control (with serum without EV depletion), while only two genes, interferon tau (*IFNT*) and *PLAC8*, were found to be downregulated in embryos compared to control (in absence of serum). Interestingly, a few genes have been studied in embryos cultured in media different to the common SOF media used in bovine embryo culture [30] (TCM199 versus DMEM), showing that the effect of oEVs on embryonic gene expression of *CX43*/*GJA1*, *GAPDH*, and *G6PD9* also depends on the medium used. However, all of the 10 genes altered due to oEV treatments in Lopera-Vasquez et al. [14,18] were found among the DEG sets in our study, which could be explained by different reasons: different oEV sources (in vitro versus in vivo origin), oviducts related to the side of ovulation and stage of the estrous cycle (ipsi- or/and contralateral oviducts; mid-luteal phase versus post-ovulatory), anatomical region of the oviduct (isthmus and ampulla versus complete oviductal fluid), EV isolation method, concentration of oEV vesicles used, the medium used for embryo culture in which the oEVs were diluted and the use of serum (without EV depletion vs. with depletion) as a supplement, and the methodology used for analyzing gene expression (RT-qPCR vs. low-input RNA-seq). The current knowledge about the effect of oEVs on embryonic development and the differences found among studies emphasizes the need for further studies to examine different variables that could affect the embryo oEV treatment (e.g., medium of embryo culture, supplements, duration of co-incubation, EV source, isolation method, and concentration) in order to establish reliable protocols that can be used to optimize in vitro embryo production in different species.

On the other hand, da Silveira et al. [31] analyzed the effect of EVs from follicular fluid during in vitro maturation and in vitro embryo culture on embryonic development and embryonic gene expression, and showed that genes involved in embryonic development (*ACSL6*, *CDH1*, *REST*) or methylation (*DNMT3A*) were altered due to follicular EV treatment during IVC. Although *CDH1*, *REST*, and *DNMT3A* have been identified in oEVs (not *ACSL6*), expression of any of these genes was altered in our study. It is worth noting that da Silveira et al. [31] found differences in embryonic gene expression between embryos supplemented with EVs from pre-ovulatory follicles and control for *ACSL6*, between treatment with EVs from follicles 3–6 mm in diameter and control for *CDH1*, and between both EV treatments and control for *REST*, indicating an important effect of the source of the EVs. The data derived from this study indicated the importance of the EV source for follicular or oviductal EVs.

In our study, we found that 25 of the 125 mRNAs upregulated in FoEV embryos compared to controls were annotated as different types of small non-coding RNAs (sncRNAs), such as small nucleolar RNA, C/D (known as SNORDs), small nucleolar RNA, H/ACA (known as SNORA), and six small nuclear RNAs, which play important roles in RNA splicing. To date, with the exception of the studies addressing the regulatory function of miRNAs in embryonic development [32,33], the regulatory function of other classes of sncRNAs in the embryo remains largely unknown. Small nucleolar RNAs form a specific class of small (60–170 nucleotides) non-coding RNAs that is best known for guiding the post-transcriptional modification of other non-coding RNAs such as ribosomal RNAs (rRNAs) and small nuclear RNAs (snRNAs) [34,35]. Additionally, snoRNAs have key regulatory functions in various other cellular processes, and their altered expression has been associated with a wide range of disorders; they have been suggested as useful diagnostic tools and biomarkers in endometrial and lung cancers [36,37]. El Hajj et al. [38] suggested that decreased SNORDs promoter region methylation in ICSI children may modulate cancer susceptibility, based on the identification of SNORDs such as *SNORD11* as being differentially methylated in ICSI versus control umbilical cord blood samples, showing a relationship to processes during early embryonic development. Interestingly, several SNORDs have been identified in EVs secreted by embryos during in vitro culture [39]. From a total of 32 snoRNAs identified, two (*SNORD110*, *SNORD81*) were exclusively present in media from viable embryos, whereas media from non-viable embryos had three exclusive snoRNAs (*SCARNA24*, *SNORD97*, *SNORD48*), indicating them as potential biomarkers of embryo viability. However, none of these snoRNAs were found in oEVs or found to be altered in the embryonic transcriptome due to oEV treatment. By contrast, the small nucleolar RNA host gene 3 (*Snhg3*), identified in oEVs, has been shown to be essential for self-renewal and pluripotency maintenance of murine embryonic stem cells (mESCs), and knockdown of *Snhg3* disrupted mouse early embryonic development [40]. Furthermore, this study showed that *Snhg3* formed a positive feedback network with *Nanog* and *Oct4* and interacted with 126 proteins in mESCs. Interestingly, the bovine *SNHG3* gene hosts the snoRNA genes *LOC112443630*, *LOC112443631*, and *LOC112443632*, belonging to the snoRNA *SNORA73* family, of which *LOC112443631* was found to be upregulated in FoEV-treated embryos. Another very recent study analyzed sncRNA expression in bovine IVF embryos during the maternal-to-zygotic transition (MZT) period and found a marked increase of sncRNAs, including snoRNAs, during the time of embryonic genome activation [41]. Based on the obtained results, the authors suggested a possible regulatory role of snoRNAs during the MZT in mammalian embryogenesis [41]. Although the role of snoRNAs in embryonic development is not very clear, the results of our and other studies call for further investigation.

To conclude this section, we would like to mention that the observed changes in embryonic gene expression as a result of embryo treatment with oEVs were analyzed after 8 days of IVC with oEVs. We hypothesize that greater transcriptome changes in embryos treated with oEVs could be found if embryos were analyzed after a shorter period of IVC, for example after a few hours. Bland et al. [42] observed changes in the T-cell transcriptome after as little as 0.5 h of exosome treatment, while other transcriptome changes were observed after 8 h of treatment. This study suggested that exosome treatment elicits a dynamic transcriptomic signature in cytotoxic T cells that becomes apparent for some clusters of genes at 0.5 h, while others needed a longer treatment period. Therefore, it is likely that a dynamic transcriptome response to oEV treatment might also be observed in embryos at different culture time points, and also depending on the embryo stage.

### 3.3. Regulation of the Embryonic Transcriptome by oEV-Derived mRNAs: mRNAs in oEVs Upregulate mRNAs in oEV-Treated Embryos

In our study, 125 genes were upregulated in embryos upon FoEV treatment during IVC. We hypothesize that this upregulation could be in part due to the incorporation of transcripts delivered by the oEVs to the embryo after uptake. An example of this oEV control of gene expression could be the higher gene expression of *SPINT2* found in FoEV-treated embryos compared to controls, and its presence in the top 500 most abundant transcripts identified in oEVs. Moreover, the functional analysis revealed that *SPINT2* is associated with “epithelial cell morphogenesis involved in placental branching” and “epithelial cell differentiation involved in embryonic placenta development”. Moreover, it has been shown that in mice, *Spint2* contributes to the appropriate development of the embryo, as indicated by *Spint2* knockout embryos showing clefting of the embryonic ectoderm, neural tube defects, and defective placental branching morphogenesis [43].

Furthermore, once the transcripts have been incorporated into the embryos, they could be translated into proteins that could regulate embryonic gene expression of the embryos, such as transcription factors. Interestingly, for one of the predicted regulators of a proportion of the FoEV-treated embryo upregulated genes, Y-box binding protein 1 (*YBX1*), the mRNA and the protein were both contained in the oEVs. YBX1 is a multifunctional protein, regulating cellular processes as a TF, involved in regulation of apoptosis, translation, cell proliferation, mRNA splicing, repair, differentiation, and stress response, and is also found in extracellular vesicles [44,45]. Recently, YBX1 has been identified as being involved in small ncRNA and mRNA sorting into exosomes [46,47]. Regarding a potential function in embryonic development, Ybx1 has been shown to play essential roles in maternal mRNA stability and early embryogenesis of zebrafish during the maternal-to-zygotic transition [48]. Additionally, *PRDX2* and *NEAT1*, genes of which the expression was also upregulated in embryos under FoEV treatment and which are targeted by transcription factor JUND, also identified in oEVs, play an important role during embryonic development. Higher expression of *PRDX2* has been associated with a greater viability of oocytes and embryos [49,50]. Moreover, a significantly lower expression of *PRDX2* was found in the first-trimester villous cytotrophoblasts of patients with recurrent miscarriage compared to healthy controls. The knockdown of *PRDX2* inhibited proliferation and increased apoptosis of trophoblast cells [51], while the lncRNA *Neat1* has been found to play a key role in corpus luteum formation and the establishment of pregnancy in a subpopulation of mice [52]. On the other hand, *PSMD4* (also known as Rpn10) was also upregulated in embryos under FoEV treatment and potentially regulated by PIN1; it has been shown to be essential for mouse development, since *Rpn10* knockout resulted in early embryonic lethality [53]. Moreover, PSMD4 protein seems to be involved in sperm–oocyte binding ability in pigs [54].

### 3.4. Regulation of the Embryonic Transcriptome by oEV-Derived miRNAs: miRNAs in oEVs Downregulate mRNAs in the Embryo

Our data analysis predicted that 62 miRNAs present in oEVs could target 57 out of the 96 obtained downregulated genes in embryos under FoEV treatment compared to control. Interestingly, some of these genes are involved in reproductive structure development processes. For example, Sp3 transcription factor (*SP3*) is involved in “trophectodermal cell differentiation” and “embryonic process involved in female pregnancy”, and can be targeted by four miRNAs identified in oEVs: miR-27a-3p, miR-484, miR-1260b, and miR-218-5p. Sp3 transcription factor is ubiquitously expressed in early embryos and, as an Sp1-like transcription factor, is a regulator of embryonic development in vertebrates [55]. Moreover, *Sp3*-/- mutant mice showed growth retardation and died at birth [56]. Another example is the gene *NANOG*, which is associated with “functional embryonic morphogenesis”, “embryonic morphogenesis”, and “gastrulation” and can be targeted by seven different miRNAs present in oEVs: miR-34a-5p, miR-34c-5p, miR-34b-3p, miR-335-5p, miR-128-3p, miR-150-5p, and miR-125b-2-3p. These two examples, among others discussed in the next section, revealed how oEV-derived miRNAs can modulate embryonic gene expression by downregulating mRNAs in the embryo.

Among the 96 genes downregulated in embryos treated with FoEVs, we found different molecules involved in toll-like receptor (TLR) pathways (TLR 2-10 pathways), as shown by the Metascape functional analysis. The TLRs are a family of innate immune system receptors which recognize various molecular patterns of microbial pathogens, inducing antimicrobial immune responses [57], and also have an important role at the maternal–fetal interface [58]. It has been proposed that exosomal miRNAs such as let-7, miR-21, and miR-29a, identified in oEVs, can act as an unconventional mode to activate TLR7 in mice and cause neurodegeneration and tumor growth and metastasis [59,60]. In the same line, milk exosomal miRNAs have been shown to decrease LPS-induced TLR4/NF-κB signaling pathway activation, reducing LPS-induced inflammation through the NF-κB pathway and inhibiting LPS-induced apoptosis via the p53 pathway [61]. Altogether, these data suggest that miRNAs present in oEVs could regulate components of TLR pathways in the embryo, modulating the maternal immune system at the maternal–fetal interface.

### 3.5. Unveiling the Potential of oEV RNA Components to Regulate Embryonic Development

To unveil the potential of oEV RNA components to regulate embryonic development, we performed an integrative analysis of mRNA and miRNA cargo identified in oEVs [19] and the embryonic transcriptome alterations induced by oEVs observed in the present study. Meta-enrichment functional analysis of these integrative data highlighted interesting functional terms highly enriched for the upregulated genes in embryos supplemented with frozen oEVs and the top 500 oEV mRNAs dataset, such as “protein methylation” and “part of non-histone protein methylation”, which is a prevalent post-translational modification and important regulator of cellular signaling and function [62]. Functional terms enriched for downregulated genes in frozen oEV embryo treatment, predicted target genes of the miRNAs in oEVs, and the top 500 oEV mRNAs dataset were related to “reproductive structure development”, among others. The parent functional term “reproductive structure development” involves child terms such as “embryonic process involved in female pregnancy”, “in utero embryonic development”, “blastocyst development”, “stem cell population maintenance”, “in utero embryonic development”, “embryo development ending in birth or egg hatching”, “trophectodermal cell differentiation”, “decidualization”, “maternal placenta development”, and “epithelial cell differentiation involved in embryonic placenta development”. Moreover, our data showed that functional terms related to both gamete formation processes such as “oocyte maturation”, “polar body extrusion after meiotic divisions”, “spindle assembly involved in meiosis”, “spermatogenesis”, “binding of sperm to zona pellucida”, “sperm–egg recognition regulation”, and “fertilization” were also enriched for downregulated genes in FoEV-treated embryos, predicted target genes of miRNAs contained in oEVs, and the top 500 oEV mRNAs. Altogether, the biological processes and pathways mentioned above bring up potential functional roles of oEVs in modulating embryonic development and contributing to successful pregnancy.

Therefore, based on the proprietary knowledge database of the IPA software, we focused the pathway analysis on the identification of genes in the list of FoEV treatment vs. Co DEGs associated with functions and processes related to embryonic development. The majority of the assigned genes were downregulated in embryos supplemented with frozen oEVs and were mainly related to apoptosis, proliferation, and viability of embryonic cells and cell lines in the IPA analysis. Many of these downregulated genes were assigned to enriched functional terms related to apoptosis, cell growth, proliferation, and embryonic morphogenesis in the Metascape analysis. One of the highly connected downregulated genes was *NANOG*, well-known as a pluripotency-sustaining factor in embryonic stem cells [63]. In rabbits, lower *NANOG* expression has been found in in vivo embryos at the 16 cell, morula, and blastocyst stages compared to the same stages in in vitro produced embryos [64]. Since *NANOG* expression is restricted to the embryoblast (EB) and is repressed in trophectoderm (TE) cells [65], this lower expression in the total blastocyst could derive from a different ratio of the number of EB and TE cells at a given stage in in-vitro-produced and in vivo embryos. It was shown in a recent study [14] that culture of bovine embryos in the presence of BOEC-derived EVs increased the number of TE cells. Altogether, this could be an indication that supplementation of embryo culture with oEVs leads to a more in-vivo-like gene expression of *Nanog*. Sirtuin 1 (SIRT1) has been shown to regulate apoptosis and *Nanog* expression in embryonic stem cells in the mouse [66]. In our study, *SIRT1* was downregulated in FoEV-treated embryos, which could have been due to miR-29b-3p, which is found in bovine oEVs [19]. In mouse embryonic stem cells, *Sirt1* has been identified as a direct target of miR-29b [67]. In addition to *Sirt1*, many more genes in the network were connected to the process “apoptosis of embryonic cell lines”. Some of the downregulated genes were typical apoptosis-promoting genes, such as *APAF1*, or genes that may have proapoptotic functions depending on the context, like *TRIM32* [68], *MAP3K1* [69], and *ATF2* [70]. One of the downregulated and highly connected genes, *MCL1*, is actually described as an anti-apoptotic factor, but is expressed in different isoforms, whereas the short version of the MCL1 protein has a proapoptotic function [71,72]. For miR-10a, targeting *APAF1* in the embryo-development-related network, anti-apoptotic effects have been shown after delivery by exosomes to follicular cells [73]. Interestingly, miR-101 has been shown recently to improve the early development of bovine somatic cell nuclear transfer embryos when overexpressed in the donor cells by increasing proliferation and vitality, and improving the early embryonic development [74].

Overall, the results revealed a network of genes and their regulatory miRNAs that suggested a mode of action of transferred oEV cargo inducing changes in embryonic gene expression which lead to a decrease in apoptosis of embryonic cells and improved embryo viability. For a variety of the identified miRNAs, a positive effect on embryonic development has been shown, for example miR-23a-3p with increased expression in outgrowth embryos in the mouse [75], and miR-21-5p with positive effects on the development of murine embryos [76].

## 4. Materials and Methods

### 4.1. Transcriptomic Analysis of Embryos by RNA Sequencing

#### 4.1.1. RNA Isolation, Low-Input Total RNA Library Preparation, and Sequencing

In-vitro-produced bovine embryos cultured in the presence or absence of oEVs for 8 days were used for transcriptomic analysis by RNA sequencing in this study. The oEVs were obtained from the oviductal fluid of bovine oviducts collected from cows at a slaughterhouse, as described in detail in our previous studies [13,19]. According to the status of the ovary, the animals were determined to be in the postovulatory stage. Pools of 8–14 embryos cultured under three different treatments during in vitro embryo culture (IVC) were used for RNA isolation: (1) embryo samples supplemented with fresh oEVs (seven replicates, a total of 74 embryos in seven embryo pools); (2) embryo samples supplemented with frozen oEVs (eight replicates, a total of 81 embryos in eight embryo pools); and (3) embryo samples without supplementation (control) (six replicates, a total of 68 embryos in six embryo pools). Total RNA from these 21 embryo pool samples was isolated using the RNeasy Micro kit (QIAGEN AG, Hombrechtikon, Switzerland) according to the manufacturer’s instructions. RNA quality and concentration were analyzed using the Agilent Bioanalyzer 2100 (Agilent Technologies (Schweiz) AG, Basel, Switzerland), NanoDrop (Thermo Fisher Scientific (Schweiz) AG, Basel, Switzerland) and Quantus Quantiflour^®^ RNA system (Promega AG, Dübendorf, Switzerland). Samples with best RNA quality and concentration were selected for the generation of RNA-seq libraries (15 libraries, five replicates/embryo treatments). For all samples, the RNA integrity number (RIN) was between 9 and 10.

For library preparation, the Ovation SOLO RNA-Seq System Kit (NuGEN Technologies, Inc. For Europe (Leek, The Netherlands) was used. Library preparation was done following the manufacturer’s instructions. In brief, aliquots of 1ng of total RNA were prepared for each embryo sample as starting material for RNA-seq library preparation. First, samples were subjected to DNase treatment and primer annealing, followed by cDNA processing and second-strand synthesis. After end repair, adapters were ligated and the first step of library amplification was performed by qPCR. To remove fragments derived from ribosomal RNAs, NuGEN’s insert-dependent adaptor cleavage (InDA-C) technology was applied in the next step. At the same time, strand selection was performed. After this step, the second library amplification and purification were performed for each sample using a universal primer and a set of barcode primers for sample multiplexing. Once RNA-seq libraries were prepared, quantitative and qualitative analyses were performed for each of the libraries using Agilent Bioanalyzer 2100 DNA High Sensitivity assays and Quantus Quantiflour^®^ ONE dsDNA system (Promega). Sequencing of the libraries was done on an Illumina HiSeq 2500 instrument (Illumina Inc., San Diego, CA, USA) at the Functional Genomics Center Zurich (FGCZ). Pooled barcoded libraries were run on two lanes of a single-end flow cell, generating between 4 and 11 million single-end reads (125 bp) per sample.

#### 4.1.2. RNA-Sequencing Data Analysis

The obtained sequence reads (Fastq files) were analyzed with an established analysis pipeline integrated in a local Galaxy installation [77] at the Animal Physiology group, ETH, Zurich. Processing, quality control, mapping, and quantification of the obtained sequences was performed as previously described [78]. The bovine genome assembly ARS-UCD1.2 (bosTau9) was used along with the corresponding GFF3 annotation file from NCBI. Based on mapping information for the reads (BAM files), a read count table for all annotated bovine genes was generated using QuasR Qcount. This count table was filtered to remove sequences with negligible read counts by using the counts per million (CPM) per sample filtering tool [79]. The mean library size and potential CPM cutoff (Counttable statistics, custom Galaxy tool) were calculated and the cutoff set to 4.21 CPM (corresponding to an average of 20 reads per library) for at least 3 out of 20 libraries. This count table was the basis for the subsequent statistical analysis.

The analysis of differential gene expression was performed using BioConductor package EdgeR [80]. Data normalization was performed based on library size (TMM normalization) [81] and with the GLM robust (estimateGLMRobustDisp) [82] function. For comparison of the experimental groups, the following contrasts were set: Frozen versus Fresh, Frozen vs. Control, and Fresh vs. Control. An adjusted *p*-value (false discovery rate, FDR) of 0.05% was used as the threshold for significance of differentially expressed genes for the Frozen vs. Control comparison. Because of the much lower number of differentially expressed genes (DEGs) obtained for the other two comparisons, the likelihood ratio (LR) of 10.81 corresponding to FDR 0.05% in the Frozen vs. Fresh comparison was used as a threshold for the other two comparisons to achieve a comparable stringency and sensitivity of the significance analysis [83]. RNA-seq data were deposited in NCBI’s Gene Expression Omnibus and are accessible through GEO Series (GSE143596, https://www.ncbi.nlm.nih.gov/geo/query/acc.cgi?acc=GSE143596).

#### 4.1.3. Data Mining and Bioinformatics Analysis

Gene symbols and Entrez Gene IDs (bovine and putative human orthologs) were mapped for all transcripts using bioinformatics custom tools integrated in a local Galaxy installation. Custom database tools (NCBI annotation mapper, Mammalian Ortholog and Annotation database, MOADb [84]) were used to assign known or putative human orthologous genes. Human gene identifiers or symbols were used for subsequent functional annotation.

To compare mRNAs altered in the presence of oEVs to oEV-derived mRNAs and potential genes targeted by miRNA-derived oEVs, Jvenn, an integrative tool for comparing lists of genes with Venn diagrams, was used [85]. Furthermore, statistical comparison of genes altered due to oEV treatment and mRNA and ncRNAs contained in oEVs was performed using GSEA software [86]. For GSEA, all identified genes in embryos were ranked based on log2-fold change and FDR (log2(fold change + 2) × −log10(FDR)) [87]. The resulting preranked gene list containing the most significantly upregulated genes at the top of the list and the most significantly downregulated genes at the bottom was compared with mRNAs and ncRNAs derived from the oEV datasets [19]. To obtain information about overrepresented biological functions and pathways for the gene sets altered due to the oEV treatments and for further comprehensive comparison with oEV-derived RNAs, the Metascape tool was used [88]. QIAGEN Ingenuity Pathway Analysis software, Winter 2019 release 2019.4 (QIAGEN Inc., https://www.qiagenbioinformatics.com/products/ingenuitypathway-analysis, [89]) was also used for functional analysis, integration, and to understand embryonic gene expression data. MIENTURNET, an interactive web tool for microRNA target enrichment analysis based on miRTarBase (an up-to-date tool for validated interactions) and TargetScan (an up-to-date tool for sequence-based miRNA target predictions) was used [90]. To identify potential transcription factors regulating embryonic gene expression, ChEA3 was used [22].

## 5. Conclusions

This study revealed a broad impact of oEVs on the embryo by providing the first high-throughput analysis of the embryonic transcriptome regulated by oEVs. Our results showed a complex embryonic molecular signature modulated by oEVs, wherein the effects of oEVs are in fact the sum of multiple effects induced by the wide range of RNA components of oEVs (mRNAs, miRNAS, SNORDs, mRNAs encoding transcription factors). By integrating data from these different oEV components and their potential effects on the embryonic transcriptome, we proposed different modes of action of oEVs on the embryo. Our study provides the basis for in-depth functional investigations of the role of specific oEV RNA cargoes (mRNAs and miRNAs) controlling early embryonic development, which could impact embryo–maternal interactions and thus have key implications for reproductive success.

## Figures and Tables

**Table 1:** Metascape functional annotation clusters for downregulated genes in embryos treated with FoEVs compared to control.

GO Term	Description	LogP	In Term/In List	Genes
GO:0034976	response to endoplasmic reticulum stress	−5.72	9/285	*APAF1,CAV1,DNAJB9,HSPA13,THBS1,EIF2AK3,SIRT1,CREBRF,STT3B,TMF1,SOCS6,RNF13,TRIM32,RYBP,NDFIP2,FBXO33*
GO:0070848	response to growth factor	−4.48	12/739	*APAF1,CAV1,EPHA2,FLT3,FOS,CCN1,MEIS2,THBS1,SHOC2,FRS2,SIRT1,FLRT3*
GO:0010942	positive regulation of cell death	−4.53	12/730	*ADAM10,APAF1,CAV1,ATF2,FOS,CCN1,MCL1,TERF1,THBS1,EIF2AK3,SIRT1,RYBP,EPHA2,TRIM32, DNAJB9,FRS2,STXBP4,TTK,MEF2A,FLT3,CREBRF,ADAMTS1,SEC24A,SGK1,NANOG*
GO:0043065	positive regulation of apoptotic process	−4.18	11/672	*ADAM10,APAF1,CAV1,ATF2,CCN1,MCL1,TERF1,THBS1,EIF2AK3,SIRT1,RYBP*
GO:0032570	response to progesterone	−3.13	3/45	*CAV1,FOS,THBS1*
GO:0008630	intrinsic apoptotic signaling pathway in response to DNA damage	−3.12	4/104	*EPHA2,MCL1,TRIM32,SIRT1*
GO:0050673	epithelial cell proliferation	−2.72	7/446	*CAV1,ATF2,EPHA2,THBS1,FRS2,SIRT1,STXBP4*
GO:0048545	response to steroid hormone	−2.38	6/385	*CAV1,FLT3,FOS,THBS1,SIRT1,CREBRF*
GO:0010035	response to inorganic substance	−2.17	7/562	*CAV1,ATF2,FOS,MEF2A,THBS1,EIF2AK3,SIRT1*
GO:2000147	positive regulation of cell motility	−2.15	7/568	*ADAM10,CAV1,CCN1,THBS1,ADAMTS1,TRIM32,SIRT1*
GO:0001525	angiogenesis	−2.07	7/588	*CAV1,ATF2,EPHA2,CCN1,THBS1,EIF2AK3,SIRT1*
R-HSA-168898	Toll-like Receptor Cascades	−2.48	4/155	*ATF2,FOS,MEF2A,MAP3K1*
M164	PID ERBB1 DOWNSTREAM PATHWAY	−2.08	3/105	*ATF2,FOS,MAP3K1*
M105	PID TELOMERASE PATHWAY	−3.82	4/68	*FOS,SP3,TERF1,TNKS,SIRT1,MIER1,NEK7,TTK,APAF1,ATF2,THBS1,RYBP,CAB39,WASHC5*
GO:0033044	regulation of chromosome organization	−2.61	6/345	*TERF1,TTK,TNKS,SIRT1,MIER1,NEK7*
GO:0045786	negative regulation of cell cycle	−2.42	8/642	*APAF1,ATF2,TERF1,THBS1,TTK,TNKS,RYBP,CAB39*
GO:0007051	spindle organization	−2.19	4/187	*TTK,TNKS,WASHC5,NEK7*
GO:0071897	DNA biosynthetic process	−2.09	4/200	*TERF1,TNKS,SIRT1,NEK7*
GO:0030155	regulation of cell adhesion	−2.79	9/693	*ADAM10,CAV1,EPHA2,CCN1,NID1,THBS1,SOCS6,NFAT5,EMILIN2*
GO:0009612	response to mechanical stimulus	−2.84	5/210	*FOS,MEIS2,MAP3K1,THBS1,SLC38A2,TNKS,EIF2AK3,SIRT1,HSPA13*
GO:0071496	cellular response to external stimulus	−2.65	6/339	*FOS,MAP3K1,TNKS,EIF2AK3,SIRT1,SLC38A2*
GO:0009266	response to temperature stimulus	−2.55	5/244	*FOS,HSPA13,THBS1,EIF2AK3,SIRT1*
GO:0048608	reproductive structure development	−2.80	7/432	*CCN1,MME,SP3,TMF1,ADAMTS1,FRS2,SIRT1,TTK,WASHC5,APAF1,NID1*
GO:0007292	female gamete generation	−2.69	4/136	*TTK,ADAMTS1,WASHC5,SIRT1*
GO:0003006	developmental process involved in reproduction	−2.28	8/677	*CCN1,MME,SP3,TMF1,ADAMTS1,WASHC5,FRS2,SIRT1*
hsa04210	Apoptosis	−2.66	4/138	*APAF1,FOS,MCL1,EIF2AK3,PFKFB3,NANOG*
M255	PID HIF1 TFPATHWAY	−2.65	3/66	*FOS,MCL1,PFKFB3*
GO:0048598	embryonic morphogenesis	−2.66	8/586	*APAF1,EPHA2,ETS2,CCN1,SP3,FRS2,FLRT3,NANOG*
GO:0007369	gastrulation	−2.21	4/185	*EPHA2,ETS2,FRS2,NANOG*
GO:0035601	protein deacylation	−2.09	3/104	*SIRT1,ABHD17B,MIER1*
R-HSA-425407	SLC-mediated transmembrane transport	−2.50	5/251	*SLC20A1,SLC38A2,SLC39A8,SLC5A11,SLC30A7*
GO:0006970	response to osmotic stress	−2.35	3/84	*ATF2,NFAT5,CAB39*
GO:0016050	vesicle organization	−2.03	5/325	*CAV1,EPS15,TMF1,SEC24A,GOLPH3*
GO:0007229	integrin-mediated signaling pathway	−2.08	3/105	*ADAM10,CDH17,ADAMTS1*

**Table 2:** Metascape functional annotation clusters for upregulated genes in embryos treated with FoEVs compared to control.

GO Term	Description	LogP	In Term/In list	Genes
R-HSA-72766	Translation	−11.09	15/291	*MRPL58,RPL23A,RPL28,RPL39,RPS4X,RPS6,RPS12,RPS17,RPS21,RPL36,MRPS26,MRPL41,MRPS34,MRPL57,MRPL52,RNA5S1,RNA5S6,RNA5S17,AP2M1,AP2S1,PSMD4,SEM1,NUP62,CHMP2A,MVB12A,QDPR,RNH1,YWHAH,POP1,PAM16, FBH1,NUDT16,TSTD1,TSR3,KRT18,VAMP8,NDUFA13,ABHD17A,ARL13A,NDUFA7,TMA7,PDLIM7,TUBA1C*
hsa03010	Ribosome	−11.07	12/153	*RPL23A,RPL28,RPL39,RPS4X,RPS6,RPS12,RPS17,RPS21,RPL36,RNA5S1,RNA5S6,RNA5S17*
R-HSA-71291	metabolism of amino acids and derivatives	−7.62	13/372	*PSMD4,QDPR,RPL23A,RPL28,RPL39,RPS4X,RPS6,RPS12,RPS17,RPS21,SEM1,RPL36,TSTD1*
R-HSA-72312	rRNA processing	−7.31	10/205	*RPL23A,RPL28,RPL39,RPS4X,RPS6,RPS12,RPS17,RPS21,RPL36,TSR3*
GO:0006401	RNA catabolic process	−7.26	13/400	*PSMD4,RNH1,RPL23A,RPL28,RPL39,RPS4X,RPS6,RPS12,RPS17,RPS21,POP1,RPL36,NUDT16*
GO:0043043	peptide biosynthetic process	−7.15	17/740	*MRPL58,NDUFA7,RPL23A,RPL28,RPL39,RPS4X,RPS6,RPS12,RPS17,RPS21,RPL36,TMA7,MRPS26,MRPL41,MRPS34, MRPL57,MRPL52*
GO:0072594	establishment of protein localization to organelle	−5.70	13/549	*RPL23A,RPL28,RPL39,RPS4X,RPS6,RPS12,RPS17,RPS21,YWHAH,NUP62,RPL36,PAM16,NDUFA13*
GO:0006605	protein targeting	−5.19	11/432	*RPL23A,RPL28,RPL39,RPS4X,RPS6,RPS12,RPS17,RPS21,RPL36,PAM16,NDUFA13*
hsa00190	oxidative phosphorylation	−4.39	6/133	*ATP5MC1,NDUFA7,NDUFB7,COX17,NDUFA13,NDUFB11*
GO:0007005	mitochondrion organization	−4.36	11/531	*ATP5MC1,NDUFA7,NDUFB7,SPG7,YWHAH,HIP1R,COX17,PAM16,NDUFA13,CYCS,NDUFB11*
GO:0046034	ATP metabolic process	−3.99	8/305	*ATP5MC1,NDUFA7,NDUFB7,TMSB4X,NUP62,NDUFA13,CYCS,NDUFB11*
GO:0032981	mitochondrial respiratory chain complex I assembly	−3.62	4/64	*NDUFA7,NDUFB7,NDUFA13,NDUFB11*
GO:0045333	cellular respiration	−3.50	6/193	*ETFB,NDUFA7,NDUFB7,NDUFA13,CYCS,NDUFB11*
R-HSA-5368287	mitochondrial translation	−5.28	6/93	*MRPL58,MRPS26,MRPL41,MRPS34,MRPL57,MRPL52*
GO:0140053	mitochondrial gene expression	−4.92	7/162	*MRPL58,NDUFA7,MRPS26,MRPL41,MRPS34,MRPL57,MRPL52*
R-HSA-199992	trans-Golgi network vesicle budding	−4.63	5/72	*FTH1,FTL,VAMP8,HIP1R,CHMP2A,AP2M1,AP2S1,YWHAH,TUBA1C,MVB12A,SLC39A4,COX17*
R-HSA-199991	membrane trafficking	−3.07	10/634	*AP2M1,AP2S1,FTH1,FTL,YWHAH,VAMP8,HIP1R,CHMP2A,TUBA1C,MVB12A*
R-HSA-5653656	vesicle-mediated transport	−2.87	10/673	*AP2M1,AP2S1,FTH1,FTL,YWHAH,VAMP8,HIP1R,CHMP2A,TUBA1C,MVB12A*
R-HSA-8876725	protein methylation	−4.03	3/19	*ETFB,METTL22,EEF2KMT*
R-HSA-195721	signaling by WNT	−2.32	6/330	*AP2M1,AP2S1,LRP5,PSMD4,SEM1,H2AC19*
R-HSA-8856828	clathrin-mediated endocytosis	−2.29	4/146	*AP2M1,AP2S1,VAMP8,HIP1R*
GO:0002478	antigen processing and presentation of exogenous peptide antigen	−2.02	4/175	*AP2M1,AP2S1,PSMD4,VAMP8*
GO:0022613	ribonucleoprotein complex biogenesis	−3.87	10/502	*RMRP,RNU2-1,RPL23A,RPS6,RPS17,RPS21,RNU4-2,RNU1-1,TSR3,NUDT16,POP1,PAM16,FBH1*
GO:0042254	ribosome biogenesis	−3.28	7/297	*RMRP,RPL23A,RPS6,RPS17,RPS21,TSR3,NUDT16*
GO:0034470	ncRNA processing	−2.63	7/384	*RMRP,RPS6,RPS17,RPS21,POP1,TSR3,NUDT16*
GO:1902686	mitochondrial outer membrane permeabilization involved in programmed cell death	−2.50	3/62	*SPG7,YWHAH,HIP1R*
GO:1905710	positive regulation of membrane permeability	−2.42	3/66	*SPG7,YWHAH,HIP1R*
GO:0097190	apoptotic signaling pathway	−2.11	8/602	*KRT18,PRDX2,YWHAH,HIP1R,BEX3,PAM16,NDUFA13,FBH1*
GO:0042058	regulation of epidermal growth factor receptor signaling pathway	−3.13	4/86	*HIP1R,NUP62,RNF126,MVB12A*
GO:0038127	ERBB signaling pathway	−2.32	4/143	*HIP1R,NUP62,RNF126,MVB12A*
GO:0030837	negative regulation of actin filament polymerization	−2.58	3/58	*CAPG,TMSB4X,HIP1R,CHMP2A*
GO:0051494	negative regulation of cytoskeleton organization	−2.27	4/148	*CAPG,TMSB4X,HIP1R,CHMP2A*
R-HSA-8953897	cellular responses to external stimuli	−2.46	8/525	*PSMD4,PRDX2,SEM1,NUP62,CHMP2A,CYCS,TUBA1C,H2AC19,VAMP8,YWHAH,SLC39A4*
R-HSA-2262752	cellular responses to stress	−2.37	7/429	*PSMD4,PRDX2,SEM1,NUP62,CYCS,TUBA1C,H2AC19*
R-HSA-109581	apoptosis	−2.01	4/176	*PSMD4,YWHAH,SEM1,CYCS*
R-HSA-5619115	disorders of transmembrane transporters	−2.01	4/176	*PSMD4,SEM1,NUP62,SLC39A4*

**Table 3:** Metascape functional annotation clusters for downregulated genes in embryos treated with FeEVs compared to control.

GO Term	Description	LogP	In Term/In List	Genes
GO:0051384	response to glucocorticoid	−4.23	3/146	*FLT3,FOS,GPR83,CTSL*
GO:0048545	response to steroid hormone	−2.99	3/385	*FLT3,FOS,GPR83*
GO:0032870	cellular response to hormone stimulus	−2.25	3/703	*CTSL,FLT3,FOS*
GO:0002521	leukocyte differentiation	−3.90	4/516	*EPHA2,FLT3,FOS,DNAJB9*
GO:0070848	response to growth factor	−3.30	4/739	*EPHA2,FLT3,FOS,CCN1,ZNF568*
GO:0070372	regulation of ERK1 and ERK2 cascade	−3.29	3/304	*EPHA2,FLT3,CCN1*
GO:0048568	embryonic organ development	−2.86	3/429	*EPHA2,CCN1,ZNF568*
GO:0048598	embryonic morphogenesis	−2.47	3/586	*EPHA2,CCN1,ZNF568*
GO:0043009	chordate embryonic development	−2.38	3/629	*EPHA2,CCN1,ZNF568*
GO:0009792	embryonic development ending in birth or egg hatching	−2.35	3/646	*EPHA2,CCN1,ZNF568*
GO:0071363	cellular response to growth factor stimulus	−2.24	3/709	*FLT3,FOS,CCN1*
GO:0043408	regulation of MAPK cascade	−2.13	3/774	*EPHA2,FLT3,CCN1*

**Table 4:** Metascape functional annotation clusters for upregulated genes in embryos treated with FeEVs compared to control.

GO Term	Description	Genes
GO:0006357	regulation of transcription by RNA polymerase II	*ZNF532, ZFP1, ZNF709*
GO:0006366	transcription by RNA polymerase II	*ZNF532, ZFP1, ZNF709*
GO:0006355	regulation of transcription, DNA-templated	*ZNF532, ZFP1, ZNF709*
GO:0051573	negative regulation of histone H3-K9 methylation	*DNMT3B*
GO:0090116	C-5 methylation of cytosine	*DNMT3B*
GO:0051571	positive regulation of histone H3-K4 methylation	*DNMT3B*
GO:0006853	carnitine shuttle	*CPT1A*
GO:0032000	positive regulation of fatty acid beta-oxidation	*CPT1A*
GO:1902001	fatty acid transmembrane transport	*CPT1A*
GO:0070981	L-asparagine biosynthetic process	*ASNS*
GO:0006529	asparagine biosynthetic process	*ASNS*
GO:0070453	regulation of heme biosynthetic process	*SRRD*
GO:1901463	regulation of tetrapyrrole biosynthetic process	*SRRD*
GO:1901401	regulation of tetrapyrrole metabolic process	*SRRD*
GO:1901647	positive regulation of synoviocyte proliferation	*NEAT1*
GO:1901645	regulation of synoviocyte proliferation	*NEAT1*
GO:0002941	synoviocyte proliferation	*NEAT1*
GO:0051058	negative regulation of small GTPase mediated signal transduction	*CGNL1*
GO:0051056	regulation of small GTPase mediated signal transduction	*CGNL1*
GO:0007015	actin filament organization	*CGNL1*
GO:0006614	SRP-dependent cotranslational protein targeting to membrane	*RPL23A*
GO:0006613	cotranslational protein targeting to membrane	*RPL23A*
GO:0000184	nuclear-transcribed mRNA catabolic process, nonsense-mediated decay	*RPL23A*

**Table 5:** Metascape functional annotation clusters for downregulated genes in embryos treated with FoEVs compared to FeEVs.

GO Term	Description	LogP	In Term/In List	Genes
R-HSA-199991	membrane trafficking	−2.48	3/634	*PAFAH1B2,DENND4C,SBF2*
R-HSA-5653656	vesicle-mediated transport	−2.41	3/673	*PAFAH1B2,DENND4C,SBF2*

**Table 6:** Metascape functional annotation clusters for upregulated genes in embryos treated with FoEVs compared to FeEVs.

GO Term	Description	LogP	In Term/In List	Genes
hsa03010	ribosome	−9.18	8/153	*RPL39,RPS4X,RPS6,RPS12,MRPL36,RNA5S1,RNA5S6,RNA5S17,DDX49,TUBA4A,PDLIM7,PDZD11*
R-HSA-6791226	major pathway of rRNA processing in the nucleolus and cytosol	−4.49	5/185	*RPL39,RPS4X,RPS6,RPS12,DDX49*
R-HSA-72312	rRNA processing	−4.27	5/205	*RPL39,RPS4X,RPS6,RPS12,DDX49*
GO:0006614	SRP-dependent cotranslational protein targeting to membrane	−4.25	4/105	*RPL39,RPS4X,RPS6,RPS12*
R-HSA-927802	nonsense-mediated decay (NMD)	−4.09	4/115	*RPL39,RPS4X,RPS6,RPS12*
GO:0045047	protein targeting to ER	−4.05	4/118	*RPL39,RPS4X,RPS6,RPS12*
R-HSA-2408522	selenoamino acid metabolism	−4.05	4/118	*RPL39,RPS4X,RPS6,RPS12*
GO:0000184	nuclear-transcribed mRNA catabolic process, nonsense-mediated decay	−4.02	4/120	*RPL39,RPS4X,RPS6,RPS12*
GO:0072599	establishment of protein localization to endoplasmic reticulum	−3.99	4/122	*RPL39,RPS4X,RPS6,RPS12*
GO:0070972	protein localization to endoplasmic reticulum	−3.68	4/147	*RPL39,RPS4X,RPS6,RPS12*
R-HSA-72649	translation initiation complex formation	−3.68	3/58	*RPS4X,RPS6,RPS12*
R-HSA-72702	ribosomal scanning and start codon recognition	−3.68	3/58	*RPS4X,RPS6,RPS12*
R-HSA-72766	translation	−3.56	5/291	*RPL39,RPS4X,RPS6,RPS12,MRPL36*
GO:0006413	translational initiation	−3.23	4/193	*RPL39,RPS4X,RPS6,RPS12*
GO:0006612	protein targeting to membrane	−3.16	4/202	*RPL39,RPS4X,RPS6,RPS12*
GO:0000956	nuclear-transcribed mRNA catabolic process	−3.10	4/209	*RPL39,RPS4X,RPS6,RPS12*
GO:0090150	establishment of protein localization to membrane	−2.36	4/334	*RPL39,RPS4X,RPS6,RPS12*
R-HSA-71291	metabolism of amino acids and derivatives	−2.20	4/372	*RPL39,RPS4X,RPS6,RPS12*
GO:0006401	RNA catabolic process	−2.09	4/400	*RPL39,RPS4X,RPS6,RPS12*
GO:0022613	ribonucleoprotein complex biogenesis	−4.25	7/502	*RMRP,RNU2-1,RPS6,RNU4-2,RNU1-1,DDX49,MRPL36*
GO:0042254	ribosome biogenesis	−2.55	4/297	*RMRP,RPS6,DDX49,MRPL36*

## Supplementary Materials

Supplementary materials can be found at https://www.mdpi.com/1422-0067/21/4/1303/s1. Supplementary Data S1. Transcriptomic data of embryos under different oEV treatments versus controls and data derived from integrative analysis with oEVs. Contains: Table S1. All identified transcripts in all embryos examined under different in vitro culture treatments. Table S2. Differentially expressed genes (DEGs) in embryos for the FoEV treatment vs. Co comparison. Table S3. Differentially expressed genes (DEGs) in embryos for the FeEV treatment vs. Co comparison. Table S4. Differentially expressed genes (DEGs) in embryos for the FoEV vs. FeEV treatment comparison. Table S5. Upregulated genes in FoEV-treated vs. Co embryos. Table S6. Downregulated genes in FoEV-treated vs. Co embryos. Table S7. All mRNAs identified in oEVs. Table S8. Top 500 most abundant mRNAs in oEVs. Table S9. Data derived from Venn diagram comparisons in Figure 5. Table S10. Data used for GSEA analysis in Figure 5. Table S11. Data derived from GSEA analysis for the Top 500 mRNAs in oEVs in Figure 5. Table S12. Data derived from GSEA analysis for the ncRNAs in oEVs in Figure 5. Table S13. Transcription factor analysis by ChEA3. Table S14. Data derived from Venn diagram comparisons in Figure 6. Table S15. Data used for Metascape circular comparisons. Supplementary Data S2. Functional analysis (FA) of transcripts up- or downregulated in embryos under different oEV treatments compared to control. Contains: Table S1. FA of transcripts downregulated in FoEV-treated embryos versus control. Table S2. FA of transcripts upregulated in FoEV-treated embryos versus control. Table S3. FA of transcripts downregulated in FeEV-treated embryos versus control. Table S4. FA of transcripts upregulated in FeEV-treated embryos versus control. Table S5. FA of transcripts downregulated in FoEV-treated versus FeEV-treated embryos. Table S6. FA of transcripts upregulated in FoEV-treated versus FeEV-treated embryos. Table S7 and Table S8. FA of transcripts included in Metascape circular analysis. Table S9 and Table S10. Molecules and pathways derived from IPA analysis and interaction pathways.

## Abbreviations

EVs	Extracellular vesicles
oEVs	Oviductal extracellular vesicles
FeEVs	Fresh oEVs
FoEVs	Frozen oEVs
DEGs	Differentially expressed genes
IVC	In vitro embryo culture

## References

[1] Maas S.L.N., Breakefield X.O., Weaver A.M. (2017). Extracellular Vesicles: Unique Intercellular Delivery Vehicles. Trends Cell Biol..

[2] Simons M., Raposo G. (2009). Exosomes—Vesicular carriers for intercellular communication. Curr. Opin. Cell Biol..

[3] Raposo G., Stoorvogel W. (2013). Extracellular vesicles: Exosomes, microvesicles, and friends. J. Cell Biol..

[4] Van der Pol E., Boing A.N., Harrison P., Sturk A., Nieuwland R. (2012). Classification, functions, and clinical relevance of extracellular vesicles. Pharmacol. Rev..

[5] Yanez-Mo M., Siljander P.R., Andreu Z., Zavec A.B., Borras F.E., Buzas E.I., Buzas K., Casal E., Cappello F., Carvalho J. (2015). Biological properties of extracellular vesicles and their physiological functions. J. Extracell. Vesicles.

[6] Gyorgy B., Szabo T.G., Pasztoi M., Pal Z., Misjak P., Aradi B., Laszlo V., Pallinger E., Pap E., Kittel A. (2011). Membrane vesicles, current state-of-the-art: Emerging role of extracellular vesicles. Cell. Mol. Life Sci..

[7] Tannetta D., Dragovic R., Alyahyaei Z., Southcombe J. (2014). Extracellular vesicles and reproduction-promotion of successful pregnancy. Cell. Mol. Immunol..

[8] Barkalina N., Jones C., Wood M.J., Coward K. (2015). Extracellular vesicle-mediated delivery of molecular compounds into gametes and embryos: Learning from nature. Hum. Reprod. Update.

[9] Saadeldin I.M., Oh H.J., Lee B.C. (2015). Embryonic-maternal cross-talk via exosomes: Potential implications. Stem Cells Cloning Adv. Appl..

[10] Ng Y.H., Rome S., Jalabert A., Forterre A., Singh H., Hincks C.L., Salamonsen L.A. (2013). Endometrial exosomes/microvesicles in the uterine microenvironment: A new paradigm for embryo-endometrial cross talk at implantation. PLoS ONE.

[11] Al-Dossary A.A., Strehler E.E., Martin-Deleon P.A. (2013). Expression and secretion of plasma membrane Ca^2+^-ATPase 4a (PMCA4a) during murine estrus: Association with oviductal exosomes and uptake in sperm. PLoS ONE.

[12] Alminana C., Bauersachs S. (2019). Extracellular Vesicles in the Oviduct: Progress, Challenges and Implications for the Reproductive Success. Bioengineering.

[13] Alminana C., Corbin E., Tsikis G., Alcantara-Neto A.S., Labas V., Reynaud K., Galio L., Uzbekov R., Garanina A.S., Druart X. (2017). Oviduct extracellular vesicles protein content and their role during oviduct-embryo cross-talk. Reproduction.

[14] Lopera-Vasquez R., Hamdi M., Fernandez-Fuertes B., Maillo V., Beltran-Brena P., Calle A., Redruello A., Lopez-Martin S., Gutierrez-Adan A., Yanez-Mo M. (2016). Extracellular Vesicles from BOEC in In Vitro Embryo Development and Quality. PLoS ONE.

[15] Lange-Consiglio A., Perrini C., Albini G., Modina S., Lodde V., Orsini E., Esposti P., Cremonesi F. (2017). Oviductal microvesicles and their effect on in vitro maturation of canine oocytes. Reproduction.

[16] Ferraz M., Carothers A., Dahal R., Noonan M.J., Songsasen N. (2019). Oviductal extracellular vesicles interact with the spermatozoon’s head and mid-piece and improves its motility and fertilizing ability in the domestic cat. Sci. Rep..

[17] Alcantara-Neto A.S., Fernandez-Rufete M., Corbin E., Tsikis G., Uzbekov R., Garanina A.S., Coy P., Alminana C., Mermillod P. (2019). Oviduct fluid extracellular vesicles regulate polyspermy during porcine invitro fertilisation. Reprod. Fertil. Dev..

[18] Lopera-Vasquez R., Hamdi M., Maillo V., Gutierrez-Adan A., Bermejo-Alvarez P., Ramirez M.A., Yanez-Mo M., Rizos D. (2017). Effect of bovine oviductal extracellular vesicles on embryo development and quality in vitro. Reprod. Fertil. Dev..

[19] Alminana C., Tsikis G., Labas V., Uzbekov R., da Silveira J.C., Bauersachs S., Mermillod P. (2018). Deciphering the oviductal extracellular vesicles content across the estrous cycle: Implications for the gametes-oviduct interactions and the environment of the potential embryo. BMC Genom..

[20] Gatien J., Mermillod P., Tsikis G., Bernardi O., Janati Idrissi S., Uzbekov R., Le Bourhis D., Salvetti P., Alminana C., Saint-Dizier M. (2019). Metabolomic Profile of Oviductal Extracellular Vesicles across the Estrous Cycle in Cattle. Int. J. Mol. Sci..

[21] Subramanian A., Kuehn H., Gould J., Tamayo P., Mesirov J.P. (2007). GSEA-P: A desktop application for Gene Set Enrichment Analysis. Bioinformatics.

[22] Keenan A.B., Torre D., Lachmann A., Leong A.K., Wojciechowicz M.L., Utti V., Jagodnik K.M., Kropiwnicki E., Wang Z., Ma’ayan A. (2019). ChEA3: Transcription factor enrichment analysis by orthogonal omics integration. Nucleic Acids Res..

[23] Sokolova V., Ludwig A.K., Hornung S., Rotan O., Horn P.A., Epple M., Giebel B. (2011). Characterisation of exosomes derived from human cells by nanoparticle tracking analysis and scanning electron microscopy. Colloids Surf. B Biointerfaces.

[24] Sarker S., Scholz-Romero K., Perez A., Illanes S.E., Mitchell M.D., Rice G.E., Salomon C. (2014). Placenta-derived exosomes continuously increase in maternal circulation over the first trimester of pregnancy. J. Transl. Med..

[25] Bosch S., de Beaurepaire L., Allard M., Mosser M., Heichette C., Chretien D., Jegou D., Bach J.M. (2016). Trehalose prevents aggregation of exosomes and cryodamage. Sci. Rep..

[26] Maroto R., Zhao Y., Jamaluddin M., Popov V.L., Wang H., Kalubowilage M., Zhang Y., Luisi J., Sun H., Culbertson C.T. (2017). Effects of storage temperature on airway exosome integrity for diagnostic and functional analyses. J. Extracell. Vesicles.

[27] Teng X., Chen L., Chen W., Yang J., Yang Z., Shen Z. (2015). Mesenchymal Stem Cell-Derived Exosomes Improve the Microenvironment of Infarcted Myocardium Contributing to Angiogenesis and Anti-Inflammation. Cell. Physiol. Biochem..

[28] Zhou H., Yuen P.S., Pisitkun T., Gonzales P.A., Yasuda H., Dear J.W., Gross P., Knepper M.A., Star R.A. (2006). Collection, storage, preservation, and normalization of human urinary exosomes for biomarker discovery. Kidney Int..

[29] Jayachandran M., Miller V.M., Heit J.A., Owen W.G. (2012). Methodology for isolation, identification and characterization of microvesicles in peripheral blood. J. Immunol. Methods.

[30] Holm P., Booth P.J., Schmidt M.H., Greve T., Callesen H. (1999). High bovine blastocyst development in a static in vitro production system using SOFaa medium supplemented with sodium citrate and myo-inositol with or without serum-proteins. Theriogenology.

[31] Da Silveira J.C., Andrade G.M., Del Collado M., Sampaio R.V., Sangalli J.R., Silva L.A., Pinaffi F.V.L., Jardim I.B., Cesar M.C., Nogueira M.F.G. (2017). Supplementation with small-extracellular vesicles from ovarian follicular fluid during in vitro production modulates bovine embryo development. PLoS ONE.

[32] Gross N., Kropp J., Khatib H. (2017). MicroRNA Signaling in Embryo Development. Biology.

[33] Liang J., Wang S., Wang Z. (2017). Role of microRNAs in embryo implantation. Reprod. Biol. Endocrinol..

[34] Matera A.G., Terns R.M., Terns M.P. (2007). Non-coding RNAs: Lessons from the small nuclear and small nucleolar RNAs. Nat. Rev. Mol. Cell Biol..

[35] Bratkovic T., Rogelj B. (2011). Biology and applications of small nucleolar RNAs. Cell. Mol. Life Sci..

[36] Ravo M., Cordella A., Rinaldi A., Bruno G., Alexandrova E., Saggese P., Nassa G., Giurato G., Tarallo R., Marchese G. (2015). Small non-coding RNA deregulation in endometrial carcinogenesis. Oncotarget.

[37] Liao J., Yu L., Mei Y., Guarnera M., Shen J., Li R., Liu Z., Jiang F. (2010). Small nucleolar RNA signatures as biomarkers for non-small-cell lung cancer. Mol. Cancer.

[38] El Hajj N., Haertle L., Dittrich M., Denk S., Lehnen H., Hahn T., Schorsch M., Haaf T. (2017). DNA methylation signatures in cord blood of ICSI children. Hum. Reprod..

[39] Mellisho E.A., Briones M.A., Velasquez A.E., Cabezas J., Castro F.O., Rodriguez-Alvarez L. (2019). Extracellular vesicles secreted during blastulation show viability of bovine embryos. Reproduction.

[40] Lu W., Yu J., Shi F., Zhang J., Huang R., Yin S., Songyang Z., Huang J. (2019). The long non-coding RNA Snhg3 is essential for mouse embryonic stem cell self-renewal and pluripotency. Stem Cell Res. Ther..

[41] Cuthbert J.M., Russell S.J., White K.L., Benninghoff A.D. (2019). The maternal-to-zygotic transition in bovine in vitro-fertilized embryos is associated with marked changes in small non-coding RNAsdagger. Biol. Reprod..

[42] Bland C.L., Byrne-Hoffman C.N., Fernandez A., Rellick S.L., Deng W., Klinke D.J. (2018). Exosomes derived from B16F0 melanoma cells alter the transcriptome of cytotoxic T cells that impacts mitochondrial respiration. FEBS J..

[43] Mitchell K.J., Pinson K.I., Kelly O.G., Brennan J., Zupicich J., Scherz P., Leighton P.A., Goodrich L.V., Lu X., Avery B.J. (2001). Functional analysis of secreted and transmembrane proteins critical to mouse development. Nat. Genet..

[44] Suresh P.S., Tsutsumi R., Venkatesh T. (2018). YBX1 at the crossroads of non-coding transcriptome, exosomal, and cytoplasmic granular signaling. Eur. J. Cell Biol..

[45] Eliseeva I.A., Kim E.R., Guryanov S.G., Ovchinnikov L.P., Lyabin D.N. (2011). Y-box-binding protein 1 (YB-1) and its functions. Biochemistry.

[46] Shurtleff M.J., Yao J., Qin Y., Nottingham R.M., Temoche-Diaz M.M., Schekman R., Lambowitz A.M. (2017). Broad role for YBX1 in defining the small noncoding RNA composition of exosomes. Proc. Natl. Acad. Sci. USA.

[47] Kossinova O.A., Gopanenko A.V., Tamkovich S.N., Krasheninina O.A., Tupikin A.E., Kiseleva E., Yanshina D.D., Malygin A.A., Ven’yaminova A.G., Kabilov M.R. (2017). Cytosolic YB-1 and NSUN2 are the only proteins recognizing specific motifs present in mRNAs enriched in exosomes. Biochim. Biophys. Acta Proteins Proteom..

[48] Yang Y., Wang L., Han X., Yang W.L., Zhang M., Ma H.L., Sun B.F., Li A., Xia J., Chen J. (2019). RNA 5-Methylcytosine Facilitates the Maternal-to-Zygotic Transition by Preventing Maternal mRNA Decay. Mol. Cell.

[49] Leyens G., Knoops B., Donnay I. (2004). Expression of peroxiredoxins in bovine oocytes and embryos produced in vitro. Mol. Reprod. Dev..

[50] Lee M.S., Liu C.H., Lee T.H., Wu H.M., Huang C.C., Huang L.S., Chen C.M., Cheng E.H. (2010). Association of creatin kinase B and peroxiredoxin 2 expression with age and embryo quality in cumulus cells. J. Assist. Reprod. Genet..

[51] Wu F., Tian F., Zeng W., Liu X., Fan J., Lin Y., Zhang Y. (2017). Role of peroxiredoxin2 downregulation in recurrent miscarriage through regulation of trophoblast proliferation and apoptosis. Cell Death Dis..

[52] Nakagawa S., Shimada M., Yanaka K., Mito M., Arai T., Takahashi E., Fujita Y., Fujimori T., Standaert L., Marine J.C. (2014). The lncRNA Neat1 is required for corpus luteum formation and the establishment of pregnancy in a subpopulation of mice. Development.

[53] Hamazaki J., Sasaki K., Kawahara H., Hisanaga S., Tanaka K., Murata S. (2007). Rpn10-mediated degradation of ubiquitinated proteins is essential for mouse development. Mol. Cell. Biol..

[54] Xuan B., Li Z.C., Wang Q.Y., Xu M., Chen X., Jin Y. (2018). Inhibition of PSMD4 alters ZP1 ubiquitination state and sperm-oocyte-binding ability in pigs. Reprod. Domest. Anim..

[55] Zhao C., Meng A. (2005). Sp1-like transcription factors are regulators of embryonic development in vertebrates. Dev. Growth Differ..

[56] Bouwman P., Gollner H., Elsasser H.P., Eckhoff G., Karis A., Grosveld F., Philipsen S., Suske G. (2000). Transcription factor Sp3 is essential for post-natal survival and late tooth development. EMBO J..

[57] Akira S., Takeda K., Kaisho T. (2001). Toll-like receptors: Critical proteins linking innate and acquired immunity. Nat. Immunol..

[58] Koga K., Aldo P.B., Mor G. (2009). Toll-like receptors and pregnancy: Trophoblast as modulators of the immune response. J. Obstet. Gynaecol. Res..

[59] Lehmann S.M., Kruger C., Park B., Derkow K., Rosenberger K., Baumgart J., Trimbuch T., Eom G., Hinz M., Kaul D. (2012). An unconventional role for miRNA: Let-7 activates Toll-like receptor 7 and causes neurodegeneration. Nat. Neurosci..

[60] Fabbri M., Paone A., Calore F., Galli R., Gaudio E., Santhanam R., Lovat F., Fadda P., Mao C., Nuovo G.J. (2012). MicroRNAs bind to Toll-like receptors to induce prometastatic inflammatory response. Proc. Natl. Acad. Sci. USA.

[61] Xie M.Y., Hou L.J., Sun J.J., Zeng B., Xi Q.Y., Luo J.Y., Chen T., Zhang Y.L. (2019). Porcine Milk Exosome MiRNAs Attenuate LPS-Induced Apoptosis through Inhibiting TLR4/NF-kappaB and p53 Pathways in Intestinal Epithelial Cells. J. Agric. Food Chem..

[62] Biggar K.K., Li S.S. (2015). Non-histone protein methylation as a regulator of cellular signalling and function. Nat. Rev. Mol. Cell Biol..

[63] Chambers I., Colby D., Robertson M., Nichols J., Lee S., Tweedie S., Smith A. (2003). Functional expression cloning of Nanog, a pluripotency sustaining factor in embryonic stem cells. Cell.

[64] Henderson G.R., Brahmasani S.R., Yelisetti U.M., Konijeti S., Katari V.C., Sisinthy S. (2014). Candidate gene expression patterns in rabbit preimplantation embryos developed in vivo and in vitro. J. Assist. Reprod. Genet..

[65] Kuckenberg P., Buhl S., Woynecki T., van Furden B., Tolkunova E., Seiffe F., Moser M., Tomilin A., Winterhager E., Schorle H. (2010). The transcription factor TCFAP2C/AP-2gamma cooperates with CDX2 to maintain trophectoderm formation. Mol. Cell. Biol..

[66] Han M.K., Song E.K., Guo Y., Ou X., Mantel C., Broxmeyer H.E. (2008). SIRT1 regulates apoptosis and Nanog expression in mouse embryonic stem cells by controlling p53 subcellular localization. Cell Stem Cell.

[67] Xu Z., Zhang L., Fei X., Yi X., Li W., Wang Q. (2014). The miR-29b-Sirt1 axis regulates self-renewal of mouse embryonic stem cells in response to reactive oxygen species. Cell Signal..

[68] Ryu Y.S., Lee Y., Lee K.W., Hwang C.Y., Maeng J.S., Kim J.H., Seo Y.S., You K.H., Song B., Kwon K.S. (2011). TRIM32 protein sensitizes cells to tumor necrosis factor (TNFalpha)-induced apoptosis via its RING domain-dependent E3 ligase activity against X-linked inhibitor of apoptosis (XIAP). J. Biol. Chem..

[69] Suddason T., Gallagher E. (2015). A RING to rule them all? Insights into the Map3k1 PHD motif provide a new mechanistic understanding into the diverse roles of Map3k1. Cell Death Differ..

[70] Vlahopoulos S.A., Logotheti S., Mikas D., Giarika A., Gorgoulis V., Zoumpourlis V. (2008). The role of ATF-2 in oncogenesis. Bioessays.

[71] Kim J.H., Sim S.H., Ha H.J., Ko J.J., Lee K., Bae J. (2009). MCL-1ES, a novel variant of MCL-1, associates with MCL-1L and induces mitochondrial cell death. FEBS Lett..

[72] Bae J., Leo C.P., Hsu S.Y., Hsueh A.J. (2000). MCL-1S, a splicing variant of the antiapoptotic BCL-2 family member MCL-1, encodes a proapoptotic protein possessing only the BH3 domain. J. Biol. Chem..

[73] Xiao G.Y., Cheng C.C., Chiang Y.S., Cheng W.T., Liu I.H., Wu S.C. (2016). Exosomal miR-10a derived from amniotic fluid stem cells preserves ovarian follicles after chemotherapy. Sci. Rep..

[74] Chang H.Y., Xie R.X., Zhang L., Fu L.Z., Zhang C.T., Chen H.H., Wang Z.Q., Zhang Y., Quan F.S. (2019). Overexpression of miR-101-2 in donor cells improves the early development of Holstein cow somatic cell nuclear transfer embryos. J. Dairy Sci..

[75] Kim J., Lee J., Jun J.H. (2019). Identification of differentially expressed microRNAs in outgrowth embryos compared with blastocysts and non-outgrowth embryos in mice. Reprod. Fertil. Dev..

[76] Lv C., Yu W.X., Wang Y., Yi D.J., Zeng M.H., Xiao H.M. (2018). MiR-21 in extracellular vesicles contributes to the growth of fertilized eggs and embryo development in mice. Biosci. Rep..

[77] Giardine B., Riemer C., Hardison R.C., Burhans R., Elnitski L., Shah P., Zhang Y., Blankenberg D., Albert I., Taylor J. (2005). Galaxy: A platform for interactive large-scale genome analysis. Genome Res..

[78] Zeng S., Ulbrich S.E., Bauersachs S. (2019). Spatial organization of endometrial gene expression at the onset of embryo attachment in pigs. BMC Genom..

[79] Chen Y., Mccarthy D., Robinson M., Smyth G.K. (2017). edgeR: Differential Expression Analysis of Digital Gene Expression Data User’s Guide. www.bioconductor.org/packages/release/bioc/vignettes/edgeR/inst/doc/edgeRUsersGuide.pdf.

[80] Robinson M.D., McCarthy D.J., Smyth G.K. (2010). edgeR: A Bioconductor package for differential expression analysis of digital gene expression data. Bioinformatics.

[81] Robinson M.D., Oshlack A. (2010). A scaling normalization method for differential expression analysis of RNA-seq data. Genome Biol..

[82] Zhou X., Lindsay H., Robinson M.D. (2014). Robustly detecting differential expression in RNA sequencing data using observation weights. Nucleic Acids Res..

[83] Hackstadt A.J., Hess A.M. (2009). Filtering for increased power for microarray data analysis. BMC Bioinform..

[84] Bick J.T., Zeng S., Robinson M.D., Ulbrich S.E., Bauersachs S. (2019). Mammalian Annotation Database for improved annotation and functional classification of Omics datasets from less well-annotated organisms. Database.

[85] Bardou P., Mariette J., Escudie F., Djemiel C., Klopp C. (2014). jvenn: An interactive Venn diagram viewer. BMC Bioinform..

[86] Subramanian A., Tamayo P., Mootha V.K., Mukherjee S., Ebert B.L., Gillette M.A., Paulovich A., Pomeroy S.L., Golub T.R., Lander E.S. (2005). Gene set enrichment analysis: A knowledge-based approach for interpreting genome-wide expression profiles. Proc. Natl. Acad. Sci. USA.

[87] Merkl M., Ulbrich S.E., Otzdorff C., Herbach N., Wanke R., Wolf E., Handler J., Bauersachs S. (2010). Microarray analysis of equine endometrium at days 8 and 12 of pregnancy. Biol. Reprod..

[88] Zhou Y., Zhou B., Pache L., Chang M., Khodabakhshi A.H., Tanaseichuk O., Benner C., Chanda S.K. (2019). Metascape provides a biologist-oriented resource for the analysis of systems-level datasets. Nat. Commun..

[89] Kramer A., Green J., Pollard J., Tugendreich S. (2014). Causal analysis approaches in Ingenuity Pathway Analysis. Bioinformatics.

[90] Licursi V., Conte F., Fiscon G., Paci P. (2019). MIENTURNET: An interactive web tool for microRNA-target enrichment and network-based analysis. BMC Bioinform..

